# Symbiotic exclusivity between CLOCK and TFPI2 drives stemness and immunosuppression in glioblastoma models

**DOI:** 10.1172/JCI199056

**Published:** 2026-03-17

**Authors:** Fei Zhou, Lizhi Pang, Yang Liu, Fatima Khan, Peiwen Chen

**Affiliations:** 1 Department of Cancer Sciences, Cleveland Clinic, Cleveland, Ohio, USA.; 2 Cleveland Clinic Lerner College of Medicine of Case Western Reserve University, Cleveland, Ohio, USA.; 3 Case Comprehensive Cancer Center, Cleveland, Ohio, USA.

**Keywords:** Immunology, Oncology, Adult stem cells, Cancer, Immunotherapy

## Abstract

Glioblastoma (GBM) is a highly aggressive brain tumor characterized by extensive crosstalk between glioblastoma stem cells (GSCs) and immunosuppressive microglia, with our previous work identifying CLOCK and TFPI2 as key regulators of this interaction. Here, we uncover a ‘symbiotic exclusivity’ pattern between CLOCK and TFPI2, showing that, despite mutually exclusive amplifications, they sustain symbiotic regulatory interactions in GBM. The CLOCK-BMAL1 complex transcriptionally upregulates TFPI2, while TFPI2-driven hypoxia inducible factor 1 α (HIF-1α) signaling activates nuclear factor k B (NF-kB) P65 to upregulate the CLOCK-BMAL1 complex, creating a positive feedback loop to promote stemness, immunosuppression, and tumor progression. Disrupting the CLOCK-TFPI2 interplay through dual inhibition of their downstream effectors reduces GSC stemness and immunosuppressive microglia, activates antitumor immunity, and synergizes with anti-PD1 therapy to achieve complete tumor regression in 50%–62.5% of tumor-bearing mice. This study uncovers a promising therapeutic strategy for a broader subset of patients with GBM with high expression of either CLOCK or TFPI2, and provides a framework for identifying ‘symbiotic exclusivity’ genes in cancer.

## Abstract 2

Scientists discovered two genes that cooperate to help aggressive brain tumors grow and evade immunity. Targeting both pathways boosts immune therapy and shrinks tumors in mice.

## Graphical abstract

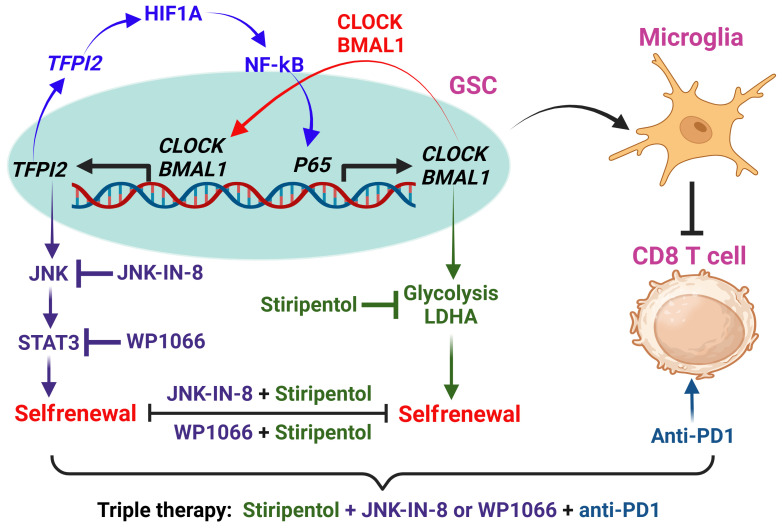

## Introduction

Glioblastoma (GBM) is the most prevalent and aggressive primary malignant brain tumor in adults (1–3). Despite great efforts that have been made on understanding the cellular and molecular mechanisms of GBM tumorigenesis, patients treated with the standard of care (including maximum surgical tumor resection followed by radiotherapy and temozolomide treatment) and immunotherapies still experience a low median overall survival ranging from 12–18 months (4, 5). Glioblastoma stem cells (GSCs) are a small subpopulation of cancer cells with self renewal, multi-potent, and tumor-initiating abilities (6, 7). GSCs harbor altered genomic stability, specific epigenetic programs, and strong symbiotic interactions with the tumor microenvironment (TME), promoting the disease progression through various mechanisms (8–12). A growing body of evidence supports the notion that the presence of GSCs in the GBM TME is the primary cause of poor prognosis, treatment resistance, and tumor recurrence (13–15). Therefore, understanding the molecular mechanisms underlying GSC stemness maintenance has become an increasingly important area of focus for GBM research (16–18).

Circadian locomotor output cycles kaput (CLOCK) and its partner basic helix-loop-helix ARNT like 1 (BMAL1, also known as ARNTL) form a heterodimeric complex that functions as a transcription factor, playing a key role in circadian rhythm regulation and contributing to the development of multiple diseases, including cancer (19). Our previous study has revealed that *CLOCK* is amplified in about 5% of GBM cases and the CLOCK-BMAL1 complex is critical for maintaining GSC stemness via intrinsically regulating glycolysis enzymes, such as the expression of lactate dehydrogenase A (LDHA), and for triggering immunosuppression by regulating the crosstalk between GSCs and microglia (20, 21). Tissue factor pathway inhibitor 2 (TFPI2), a member of the Kunitz-type serine proteinase inhibitor family, is a key regulator of extracellular matrix remodeling via inhibiting promatrix metalloproteinase 1 (MMP1) and pro-MMP13 (22). *TFPI2* was previously recognized as a tumor suppressor (23–25). Recently, we found that *TFPI2* is amplified in about 4%–6% of GBM cases, where it exhibits an important role in promoting GSC self renewal via activating the Jun N-terminal kinase–signal (JNK-signal) transducer and activator of transcription 3 (STAT3) signaling pathway and in driving immunosuppression by regulating microglia infiltration and immunosuppressive polarization (16). In GBM mouse models, depletion of CLOCK or TFPI2 in GSCs reduces GBM progression, self-renewing GSCs, and immunosuppressive microglia (16, 20, 21), highlighting CLOCK and TFPI2 as promising therapeutic targets for GBM treatment. However, the limitations for these studies are related to the genomic data showing that *CLOCK* and *TFPI2* are amplified in a small subset of patients with GBM (16, 20). Even more challenging is the fact that no specific inhibitors are currently available to target CLOCK and TFPI2.

In this study, we introduced the ‘symbiotic exclusivity’ conceptual framework to describe the relationship between CLOCK and TFPI2 in GBM, where they exhibit mutually exclusive amplification patterns but form a positive feedback loop to coordinate GSC self renewal, immunosuppression, and tumor progression. At the mechanistic level, the CLOCK-BMAL1 complex can transcriptionally upregulate TFPI2 in GSCs, and, reciprocally, TFPI2-directed hypoxia-inducible factor 1 α (HIF-1α) activates nuclear factor κ B (NF-κB) P65 to upregulate the CLOCK-BMAL1 complex, forming a positive feedback loop to promote the self renewal of GSCs and their associated immunosuppression in GBM. Due to the lack of inhibitors directly and specifically targeting CLOCK or TFPI2, our preclinical trials in this study employed a strategy to jointly target their downstream signals, LDHA and the JNK-STAT3 signaling axis. We confirmed that the dual treatment strategy disrupts the GSC-microglia interplay, remodels the TME from “cold” to “hot”, and leads to disease eradication in GBM mouse models when combined with anti-PD1 immunotherapy. These findings reveal a promising therapeutic strategy for a broader subset of patients with GBM expressing either CLOCK or TFPI2, and lay the foundation for identifying therapeutically targetable ‘symbiotic exclusivity’ patterns across cancers.

## Results

### The CLOCK-BMAL1 complex regulates TFPI2 expression in GSCs.

**Figure 1:**
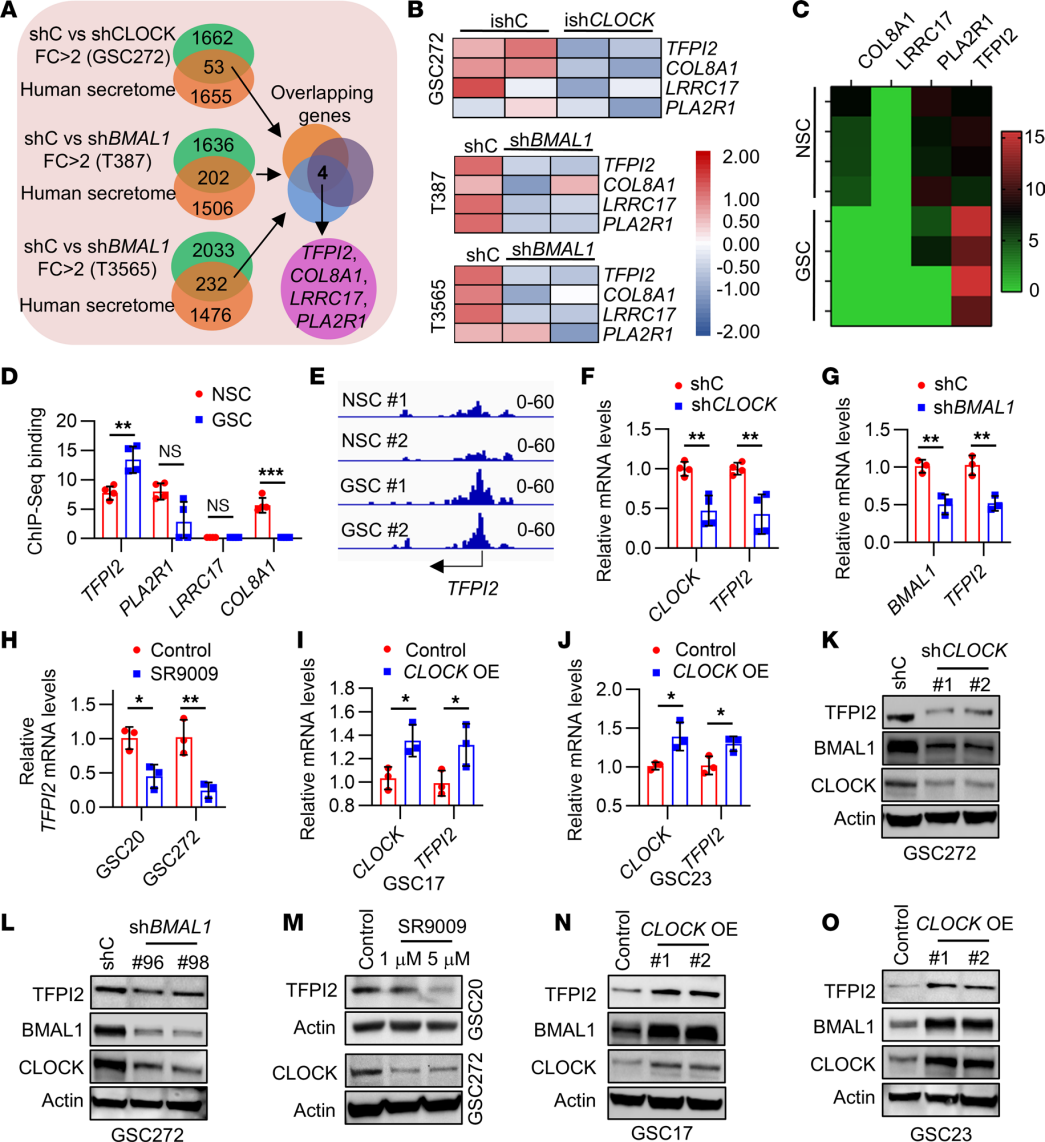
The CLOCK-BMAL1 complex regulates TFPI2 expression in GSCs. (**A**) Strategy for identification of 4 key genes encoding secreted proteins that are downregulated (fold change > 2) by CLOCK depletion in GSC272 with inducible *CLOCK* shRNA (ish*CLOCK*) versus inducible shRNA control (ishC), and by BMAL1 depletion in T387 and T3565 with *BMAL1* shRNA (sh*BMAL1*) versus shRNA control (shC). (**B**) Heat map shows the expression of *TFPI2*, *COL8A1*, *LRRC17,* and *PLA2R1* in GSC272, T387, and T3565 harboring ishC versus ish*CLOCK* and shC versus sh*BMAL1*. Red and blue indicate higher and lower expression, respectively. (**C** and **D**) ChIP-seq data analysis shows the heatmap (**C**) and quantification (**D**) of BMAL1-enriched profiles at *TFPI2*, *COL8A1*, *LRRC17,* and *PLA2R1* promotors in GSCs (T387 and T3565) and NSCs (ENSA and hNP1). *n* = 4. (**E**) ChIP-seq data analysis shows BMAL1-enriched profiles at the *TFPI2* promotor in NSC #1 and #2 (ENSA and hNP1) and GSC #1 and #2 (T387 and T3565). (**F** and **G**) RT-qPCR for *CLOCK* and *TFPI2* in control, CLOCK-depleted (**F**, *n* = 4), and BMAL1-depleted (**G**, *n* = 3) GSC272. (**H**) RT-qPCR for *TFPI2* in GSC20 and GSC272 treated with or without SR9009 (5 μmol/L). *n* = 3 independent samples. (**I** and **J**) RT-qPCR for *CLOCK* and *TFPI2* in GSC17 (**I**) or GSC23 (**J**) harboring *CLOCK* overexpression (OE). *n* = 3. (**K** and **L**) Immunoblots for CLOCK, BMAL1, and TFPI2 in GSC272 harboring shC and sh*CLOCK* (**K**), or shC and sh*BMAL1* (**L**). (**M**) Immunoblots for CLOCK and TFPI2 in GSC20 and GSC272 treated with or without SR9009 (1 and 5 μmol/L). (**N** and **O**) Immunoblots for CLOCK, BMAL1, and TFPI2 in GSC17 (**N**) or GSC23 (**O**) harboring *CLOCK* OE. Data from multiple replicates are presented as mean ± SD. *P < 0.05, **P < 0.01, ***P < 0.001, Student’s *t* test (**D** and **F**–**J**).

Our previous studies demonstrated that the CLOCK-BMAL1 complex plays a crucial role in regulating the self renewal of GSCs and their interaction with the TME (20). To better understand these processes, we analyzed the microarray profiling data from GSC272 harboring shRNA control (shC) and shRNA *CLOCK* (sh*CLOCK*) (20), RNA-seq data from GSCs (T387 and T3565) expressing shC and sh*BMAL1* (26), and intersected CLOCK- and BMAL1-regulated genes with human secretome database (27). These analyses lead to the identification of 4 genes (*TFPI2*, *PLA2R1*, *LRRC17*, and *COL8A1*) that were downregulated by depletion of the CLOCK-BMAL1 complex in GSCs (Figure 1, A and B). The CLOCK-BMAL1 complex functions as a key transcription factor regulating circadian rhythm and other cellular processes (21, 28). To investigate whether the CLOCK-BMAL1 complex can transcriptionally regulate these 4 genes in GSCs, we analyzed the BMAL1 ChIP-Seq data from human neural stem cells (NSCs) and GSCs (26). We found that *BMAL1* binding to the *TFPI2* and *COL8A1* promoters was enhanced and reduced, respectively, in GSCs compared with NSCs, while binding to the *PLA2R1* and *LRRC17* promoters remained unchanged between GSCs and NSCs (Figure 1, C–E). RT-qPCR demonstrated that inhibition of the CLOCK-BMAL1 complex using shRNA-mediated depletion of CLOCK or BMAL1, or using the treatment with SR9009, an agonist of nuclear receptors REV-ERBs that can suppress BMAL1 (29, 30), significantly reduced the expression of *TFPI2* in GBM patient-derived GSC272 and GSC20 (Figure 1, F–H) or mouse GBM tumor-derived GSCs (Supplemental Figure 1, A–D; supplemental material available online with this article; https://doi.org/10.1172/JCI199056DS1), such as 005 GSC, a line isolated from Trp53^+/−^ mouse brains with lentiviral transduction of activated AKT and H-Ras (31, 32), and QPP7 GSC, a line isolated from mouse brains harboring null alleles of *Qki*, *Pten*, and *Trp53* (33). Conversely, overexpression of CLOCK in GBM patient-derived GSC17 and GSC23 upregulated *TFPI2* expression (Figure 1, I and J). Similarly, Western blotting confirmed that shRNA-mediated depletion of CLOCK and BMAL1 and SR9009 treatment dramatically reduced the protein level of TFPI2 in human GSC272 and GSC20 (Figure 1, K–M) and mouse GSCs (Supplemental Figure 1, E–I), such as 005 GSC, QPP7 GSC, and CT2A, a GBM cell line harboring GSC-like phenotype (16, 21). Overexpression of CLOCK in GSC17 and GSC23, however, exhibited an opposite effect (Figure 1, N and O). Our previous studies have shown that olfactomedin-like 3 (OLFML3) is downstream of CLOCK in GSCs (20, 21). To investigate whether TFPI2 is regulated by the OLFML3 pathway, we depleted OLFML3 in GSC272 cells and found that this modification did not affect the expression of TFPI2 (Supplemental Figure 1J). Together, these findings indicate that TFPI2 is transcriptionally regulated by the CLOCK-BMAL1 complex in GSCs.

### CLOCK-induced GSC self renewal is regulated by TFPI2.

**Figure 2:**
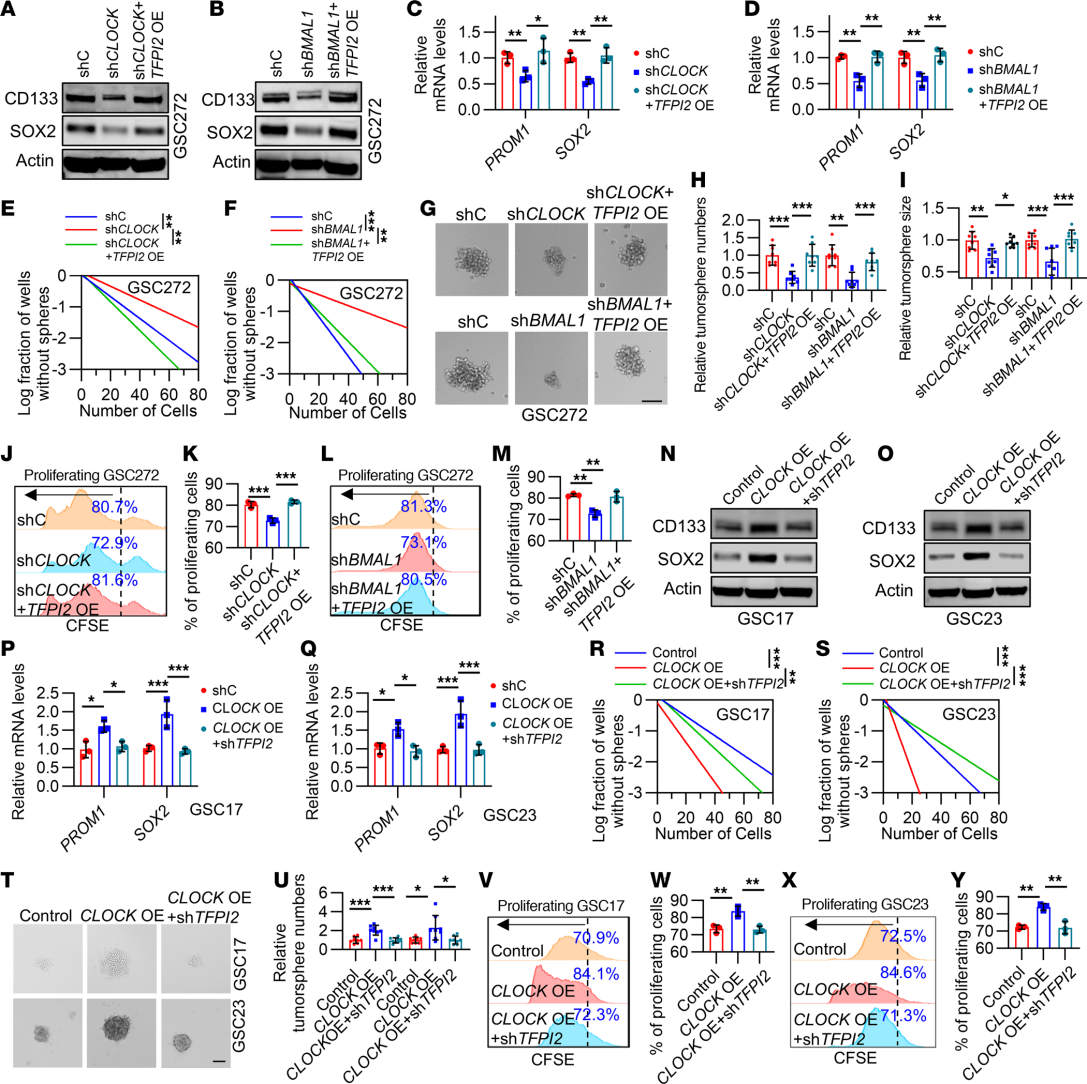
CLOCK-induced GSC self renewal is regulated by TFPI2. (**A**–**D**) Immunoblots (**A** and **B**) and RT-qPCR (**C** and **D**, *n* = 3) for CD133 (*PROM1*) and SOX2 in GSC272 harboring shRNA control (shC), CLOCK shRNA (sh*CLOCK*, **A**), or sh*BMAL1* (**B**) in the presence or absence of *TFPI2* overexpression (OE). *n* = 3. (**E** and **F**) In vitro limiting dilution assays in GSC272 harboring shC, sh*CLOCK* (**E**), or sh*BMAL1* (**F**) in the presence or absence of *TFPI2* OE. (**G**–**I**) Representative images (**G**) and quantification of relative tumorsphere number (**H**) and size (**I**) of GSC272 expressing shC, sh*CLOCK,* or sh*BMAL1* in the presence or absence of *TFPI2* OE. Scale bar: 200 μm. *n* = 8. (**J**–**M**) Representative images and quantification of proliferation in GSC272 harboring shC, sh*CLOCK* (**J** and **K**), and sh*BMAL1* (**L** and **M**) with or without *TFPI2* OE. *n* = 3. (**N**–**Q**) Immunoblots (**N** and **O**) and RT-qPCR (**P** and **Q**, *n* = 3) for CD133 (*PROM1*) and SOX2 in GSC17 and GSC23 harboring Control or *CLOCK* OE with or without sh*TFPI2*. (**R** and **S**) In vitro limiting dilution assays in GSC17 (**R**) and GSC23 (**S**) expressing Control or *CLOCK* OE with or without sh*TFPI2*. (**T** and **U**) Representative images (**T**) and quantification of relative tumorsphere number (**U**) of GSC17 or GSC23 harboring *CLOCK* OE in the presence or absence of sh*TFPI2*. Scale bar: 200 μm. *n* = 8. (**V**–**Y**) Representative images and quantification of proliferation in GSC17 (**V** and **W**) and GSC23 (**X** and **Y**) harboring *CLOCK* OE in the presence or absence of sh*TFPI2*. *n* = 3. Data from multiple replicates are presented as mean ± SD. *P < 0.05, **P < 0.01, ***P < 0.001, 1-way ANOVA test (**C**, **D**, **H**, **I**, **K**, **M**, **P**, **Q**, **U**, **W**, and **Y**), 2-way ANOVA test (**E**, **F**, **R**, and **S**).

Our previous studies revealed that CLOCK is essential for GSC stemness maintenance (20, 26). Here, we further investigated whether TFPI2 is required for CLOCK-induced GSC self renewal. Western blotting and RT-qPCR showed that CLOCK or BMAL1 depletion–induced downregulation of stemness-associated factors CD133 and SOX2 at both protein and mRNA levels was rescued by TFPI2 overexpression in human GSC272 (Figure 2, A–D), mouse QPP7 GSC, and 005 GSC (Supplemental Figure 2, A–F). Limiting dilution and tumorsphere formation assays demonstrated that TFPI2 overexpression negated the impairment of GSC self renewal induced by depletion of the CLOCK-BMAL1 complex in GSC272 (Figure 2, E–I), QPP7 GSC (Supplemental Figure 2, G–K), and 005 GSC (Supplemental Figure 2, L–O). Moreover, carboxyfluorescein diacetate succinimidyl ester (CFSE) assays demonstrated that the proliferation deficits in CLOCK- or BMAL1-depleted GSC272 (Figure 2, J–M), QPP7 GSC (Supplemental Figure 2, P–S), and 005 GSC (Supplemental Figure 2, T and U) were abolished by TFPI2 overexpression.

To further confirm this connection, we overexpressed *CLOCK* in GSC23 and GSC17 that harbor low CLOCK expression (20) and then utilized shRNA-mediated knockdown system to deplete *TFPI2*. Consistent with our previous studies showing that CLOCK overexpression upregulated the protein and mRNA levels of CD133 and SOX2, which were negated by TFPI2 knockdown (Figure 2, N–Q). Moreover, TFPI2 knockdown negated CLOCK overexpression–induced upregulation of self renewal (Figure 2, R–U, and Supplemental Figure 2, V and W) and proliferation (Figure 2, V–Y). Together, these findings highlight that TFPI2 functions as a key factor in determining the CLOCK-BMAL1 complex-induced GSC self renewal.

### Mutual exclusive nature of CLOCK and TFPI2 and its function in regulating GSC self renewal.

**Figure 3:**
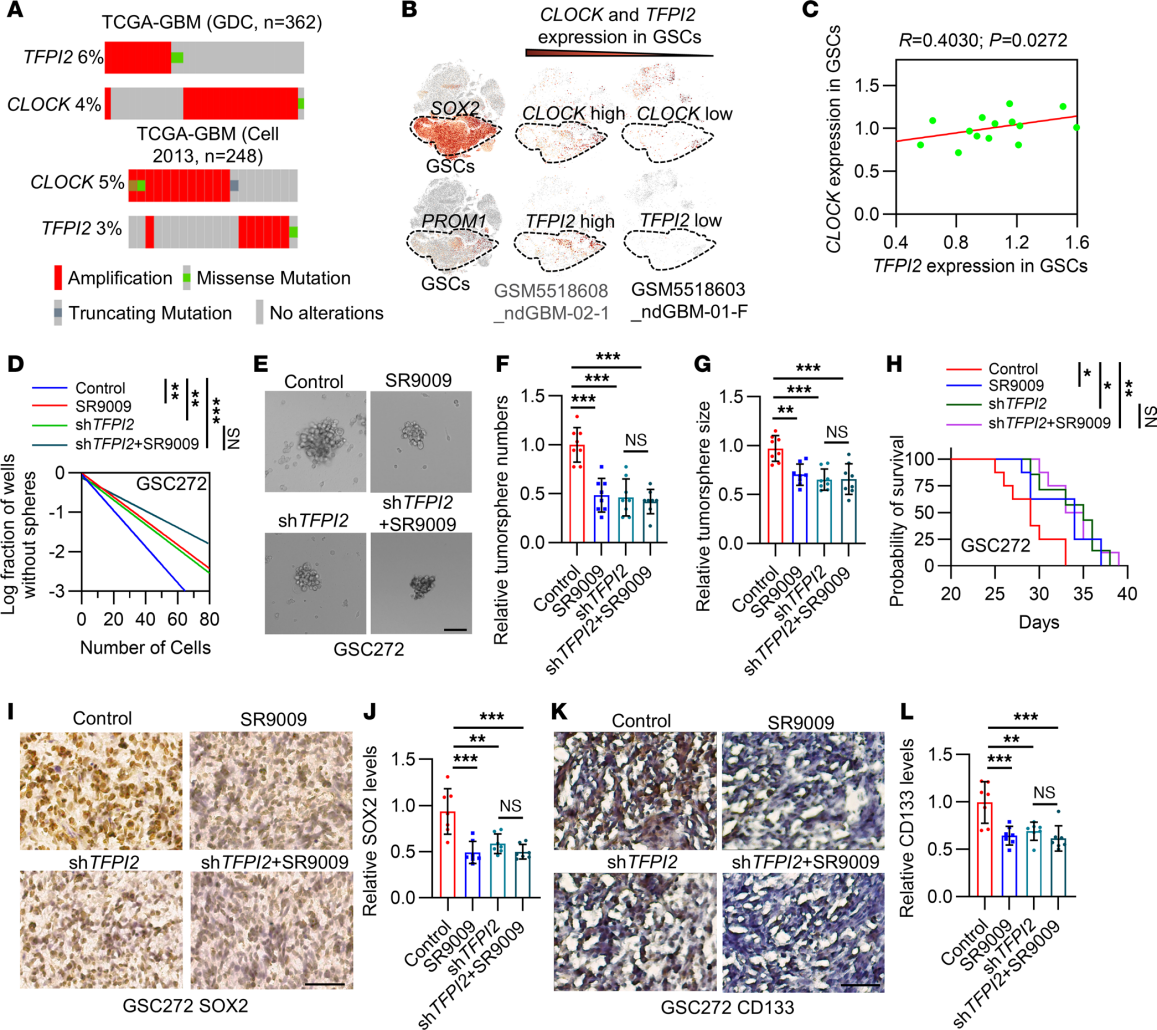
Mutual exclusivity between *CLOCK* and *TFPI2* contributes to GSC self renewal and GBM progression. (**A**) Amplification pattern *TFPI2* and *CLOCK* in TCGA GBM patient tumors from indicated datasets. (**B**) High-resolution uniform manifold approximation and projection (UMAP) showing expression of *CLOCK* and *TFPI2* in GSCs (the population of cells are highlighted in circle) of GBM patient tumors based on scRNA-Seq data (GSE182109). (**C**) Relationship between *CLOCK* and *TFPI2* expression in GSCs from GBM patient tumors based on scRNA-Seq data (GSE182109). *R* and *P* values are shown. Pearson’s correlation test. (**D**) In vitro limiting dilution assays in GSC272 harboring shRNA control (shC) and *TFPI2* shRNA (sh*TFPI2*) treated with or without SR9009 (5 μM). (**E**–**G**) Representative images (**E**) and quantification of relative tumorsphere number (**F**) and size (**G**) of GSC272 harboring shC and sh*TFPI2* and treated with or without SR9009 (5 μM). Scale bar: 200 μm. *n* = 8. (**H**) Survival curves of nude mice implanted with 2 × 10^5^ shC and sh*TFPI2* GSC272. Mice were treated with SR9009 (100 mg/kg, i.p., daily) for 10 days beginning at day 7 after orthotopic injection (*n* = 8 mice per group). (**I**–**L**) Representative images and quantification of IHC staining for SOX2 (**I** and **J**) and CD133 (**K** and **L**) in shC and sh*TFPI2* GSC272 tumors from mice treated with or without SR9009. Scale bar: 100 μm. *n* = 7. Data from multiple replicates are presented as mean ± SD. *P < 0.05, **P < 0.01, ***P < 0.001, 1-way ANOVA test (**F**, **G**, **J**, and **L**), 2-way ANOVA test (**D**), log-rank test (**H**).

Bioinformatic analysis demonstrated that *CLOCK* amplification is mutually exclusive with *TFPI2* amplification in TCGA GBM datasets (Figure 3A), suggesting that CLOCK and TFPI2 might interact with each other by sharing similar functions (34, 35). Analysis of single-cell RNA sequencing (scRNA-seq) data from GBM patient tumors (36) demonstrated that *CLOCK* and *TFPI2* were highly expressed in GSCs compared with tumors cells (Supplemental Figure 3, A–D). Correlation analysis in another scRNA-seq dataset from GBM patient tumors (37) confirmed that the expression of *CLOCK* was positively correlated with *TFPI2* in GSCs (Figure 3, B and C). These results indicate a shared role for CLOCK and TFPI2 in GSCs and point to the possibility of a positive feedback loop between them. To confirm it through experimentation, we dually inhibited CLOCK (using SR9009 or sh*CLOCK*) and TFPI2 (using sh*TFPI2*) in GSC272. Consistent with our previous studies (16, 20) showing that SR9009 treatment, sh*CLOCK*, or sh*TFPI2* reduced the self renewal (Figure 3, D–G, and Supplemental Figure 3, E–J) and proliferation (Supplemental Figure 3, K and L) and upregulated apoptosis (Supplemental Figure 3, M and N) of GSC272. However, dual inhibition of CLOCK and TFPI2 (SR9009/sh*CLOCK*+sh*TFPI2*) did not exhibit additional effects on GSC self renewal (Figure 3, D–G, and Supplemental Figure 3, E–J), proliferation (Supplemental Figure 3, K and L), and apoptosis (Supplemental Figure 3, M and N). Similar effects were observed in GSC272 tumor-bearing mice showing that the combined treatment with SR9009 and sh*TFPI2* did not affect survival, proliferation, apoptosis, and GSC stemness compared with the single treatment (Figure 3, H–L, and Supplemental Figure 3, O–R). Together, these findings suggest that CLOCK and TFPI2 promote stemness, likely by forming a positive feedback loop based on their mutual exclusivity in GBM.

### TFPI2 upregulates the CLOCK-BMAL1 complex to promote GSC self renewal and tumor growth.

**Figure 4:**
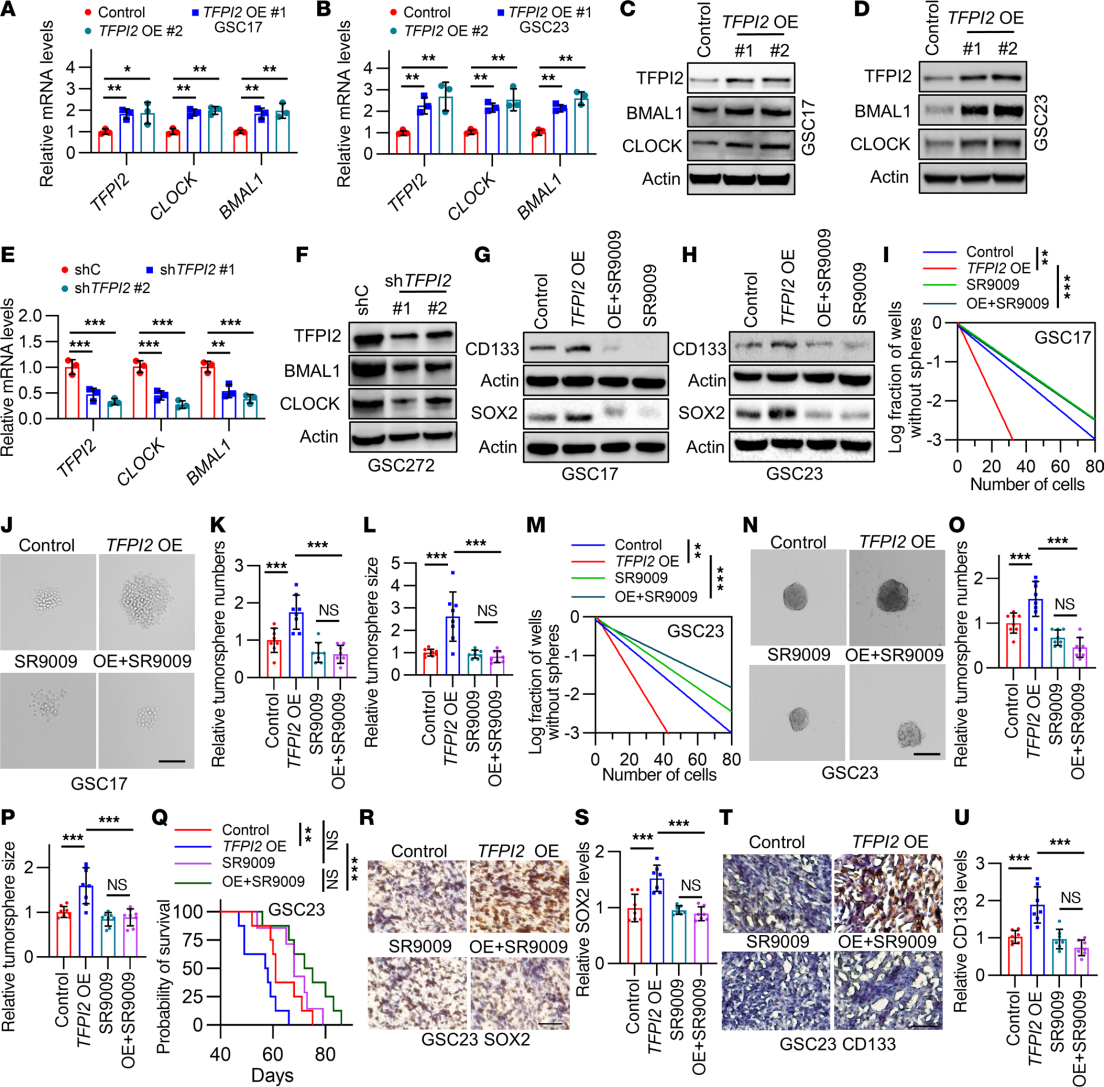
TFPI2 upregulates the CLOCK-BMAL1 complex to promote GSC self renewal and GBM progression. (**A**–**D**) RT-qPCR (**A** and **B**, *n* = 3) and immunoblots (**C** and **D**) for TFPI2, CLOCK, and BMAL1 in GSC17 and GSC23 harboring control or *TFPI2* overexpression (OE). (**E**) RT-qPCR (**E**, *n* = 3) and immunoblots (**F**) for TFPI2, CLOCK, and BMAL1 in GSC272 harboring shRNA control (shC) or *TFPI2* shRNA (sh*TFPI2*). (**G** and **H**) Immunoblots for CD133 and SOX2 in GSC17 (**G**) and GSC23 (**H**) harboring control or *TFPI2* OE and treated with or without SR9009 (5 μM). (**I**) In vitro limiting dilution assays in GSC17 harboring control or *TFPI2* OE and treated with or without SR9009 (5 μM). (**J**–**L**) Representative images (**J**) and quantification of relative tumorsphere number (**K**) and size (**L**) of GSC17 harboring control or *TFPI2* OE treated with or without SR9009 (5 μM). Scale bar: 200 μm. *n* = 8. (**M**) In vitro limiting dilution assays in GSC23 harboring control or *TFPI2* OE and treated with or without SR9009 (5 μM). (**N**–**P**) Representative images (**N**) and quantification of relative tumorsphere number (**O**) and size (**P**) of GSC23 harboring control or *TFPI2* OE treated with or without SR9009 (5 μM). Scale bar: 200 μm. *n* = 8. (**Q**) Survival curves of nude mice implanted with 2 × 10^5^ Control and *TFPI2* OE GSC23 and treated with SR9009 (100 mg/kg, i.p., daily) for 10 days beginning at day 7 (n = 8 mice per group). (**R**–**U**) Representative images and quantification of IHC staining for SOX2 (**R** and **S**) and CD133 (**T** and **U**) in Control and *TFPI2* OE GSC23 tumors from mice treated with or without SR9009. Scale bar: 100 μm. *n* = 7. Data from multiple replicates are presented as mean ± SD. *P < 0.05, **P < 0.01, ***P < 0.001, 1-way ANOVA test (**A**, **B**, **E**, **K**, **L**, **O**, **P**, **S**, and **U**), 2-way ANOVA test (**I** and **M**), log-rank test (**Q**).

To further confirm the positive feedback loop between CLOCK and TFPI2 in GSCs, we investigated whether TFPI2 can reciprocally regulate the CLOCK-BMAL1 complex in GSCs. By overexpressing TFPI2 in patient-derived GSC23 and GSC17 with relatively low TFPI2 expression (16), we found that such modification upregulated the expression of CLOCK and BMAL1 at both mRNA and protein levels (Figure 4, A–D). Conversely, shRNA-mediated depletion of TFPI2 reduced the mRNA and protein levels of CLOCK and BMAL1 in human GSC272 (Figure 4, E and F), and mouse GSCs, such as QPP7 GSC (Supplemental Figure 4, A and B), CT2A, and 005 GSC (Supplemental Figure 4, C–E), all of which harbor high TFPI2 expression (16). Next, we aimed to investigate whether TFPI2-regulated CLOCK-BMAL1 complex contributes to GSC stemness maintenance. Pharmacologic inhibition of the CLOCK-BMAL1 complex with SR9009 negated the expression of stemness-related factors SOX2 and CD133 (Figure 4, G and H, and Supplemental Figure 4F), self renewal (Figure 4, I–P), proliferation (Supplemental Figure 4, G–J), and apoptotic (Supplemental Figure 4, K–N) effects of increasing cellular TFPI2 in GSC17 and GSC23. In CLOCK-low GSC23 tumor-bearing mice, SR9009 treatment did not extend the survival, while TFPI2 overexpression shortened the survival, an effect that was abolished by the treatment with SR9009 (Figure 4Q). On the histological level, TFPI2 overexpression–induced upregulation of GSC stemness markers SOX2 and CD133 were negated by the treatment with SR9009 (Figure 4, R–U). Immunofluorescence for Ki67 and cleaved caspase 3 confirmed that SR9009 blocked the increase in proliferation and the reduction in apoptosis induced by TFPI2 overexpression (Supplemental Figure 4, O–R). Together, these findings suggest that the CLOCK-BMAL1 complex is required for TFPI2 overexpression–induced GSC self renewal and GBM progression.

### TFPI2 regulates the CLOCK-BMAL1 complex through HIF-1α–NF-κB signaling pathway in GSCs.

**Figure 5:**
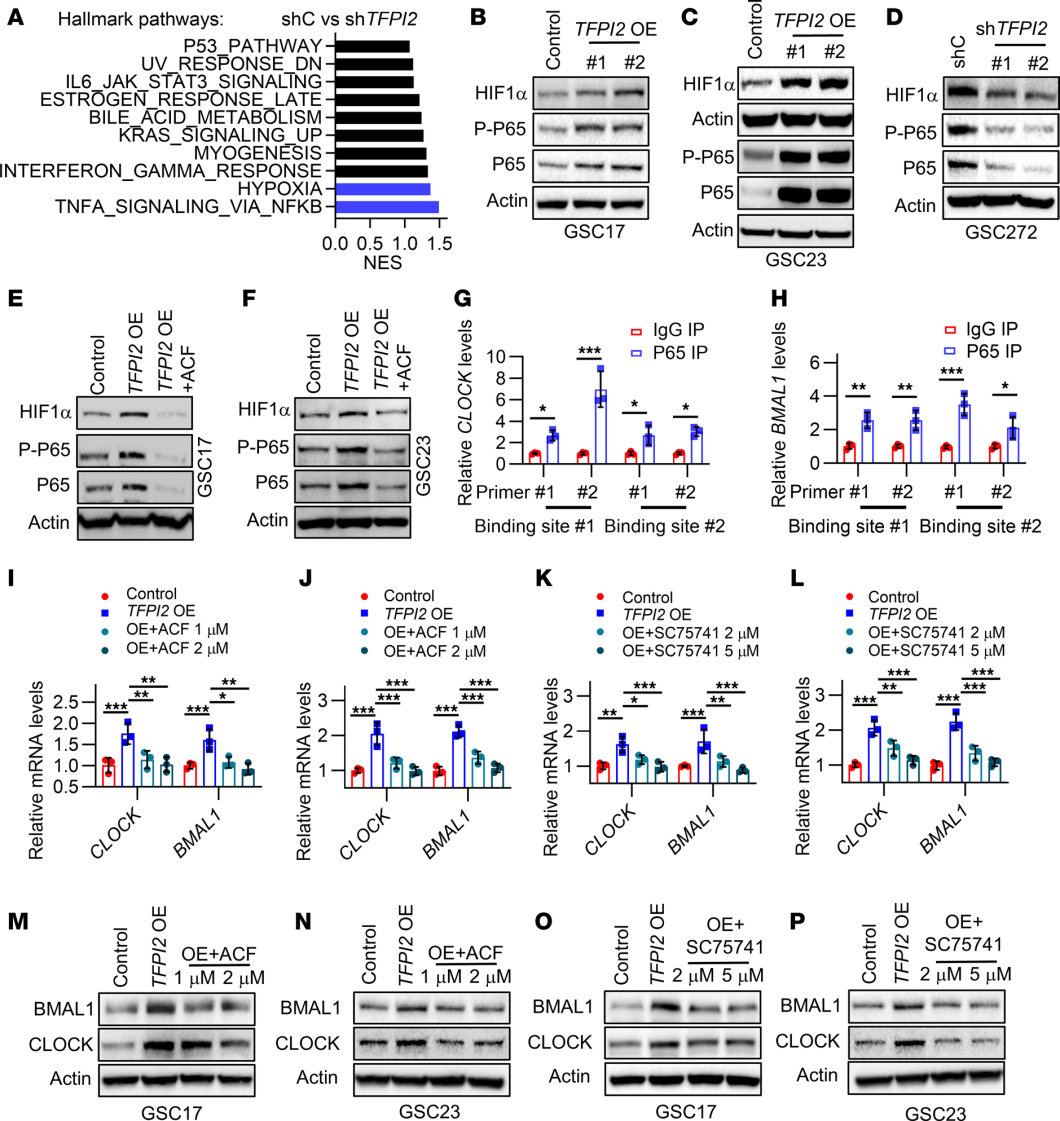
TFPI2 regulates CLOCK-BMAL1 complex through HIF-1α–NF-κB signaling in GSCs. (**A**) GSEA analysis shows the top 10 enriched hallmark pathways in shRNA control (shC) GSC272 compared with *TFPI2* shRNA (sh*TFPI2*) GSC272. Blue bars indicate the top 2 enriched signatures (*FDR* < 0.25). (**B** and **C**) Immunoblots for HIF-1α, P-P65, and P65 in cell lysates of GSC17 (**B**) and GSC23 (**C**) harboring control and *TFPI2* overexpression (OE). (**D**) Immunoblots for HIF-1α, P-P6,5 and P65 in cell lysates of shC and sh*TFPI2* GSC272. (**E** and **F**) Immunoblots for HIF-1α, P-P65, and P65 in cell lysates of GSC17 (**E**) and GSC23 (**F**) harboring control and *TFPI2* OE treated with or without HIF-1α inhibitor ACF (2 μM). (**G** and **H**) Quantification of p65 ChIP-PCR in the *CLOCK* promoter (**G**) and *BMAL1* promoter (**H**) of GSC272. IgG was used as Control. *n* = 3. (**I** and **J**) RT-qPCR for *CLOCK* and *BMAL1* in GSC17 (**I**) and GSC23 (**J**) harboring *TFPI2* OE treated with or without ACF. *n* = 3. (**K** and **L**) RT-qPCR for *CLOCK* and *BMAL1* in GSC17 (**K**) and GSC23 (**L**) harboring control or *TFPI2* OE treated with or without P65 inhibitor SC75741. *n* = 3. (**M** and **N**) Immunoblots for CLOCK and BMAL1 in cell lysates of control and *TFPI2* OE GSC17 (**M**) or GSC23 (**N**) treated with or without ACF. (**O** and **P**) Immunoblots for CLOCK and BMAL1 in cell lysates of control and *TFPI2* OE GSC17 (**O**) or GSC23 (**P**) treated with or without SC75741. Data from multiple replicates are presented as mean ± SD. *P < 0.05, **P < 0.01, ***P < 0.001, Student’s *t* test (**G** and **H**), 1-way ANOVA test (**I**–**L**).

To determine how TFPI2 regulates the CLOCK-BMAL1 complex in GSCs, we performed gene set enrichment analysis (GSEA) on hallmark pathways in GSC272 with shC versus sh*TFPI2*. The results showed that NF-κB and Hypoxia pathways were the top 2 enriched in control cells compared with TFPI2-depleted cells (Figure 5A). Western blotting results demonstrated that TFPI2 overexpression upregulated the expression of HIF-1α, NF-κB P65, and Phospho-NF-κB P65 (P-P65) in GSC17 and GSC23 (Figure 5, B and C), and the opposite effect was observed upon depletion of TFPI2 in GSC272 (Figure 5D), QPP7 GSC (Supplemental Figure 5A), and CT2A (Supplemental Figure 5B). To reveal the relationship between HIF-1α and P65, TFPI2-overexpressing GSC17 and GSC23 were treated with or without HIF-1α inhibitor acriflavine (ACF) or NF-κB P65 inhibitor SC75741. Western blotting demonstrated that TFPI2-induced P65 and P-P65 were blocked by HIF-1α inhibition (Figure 5, E and F). However, NF-κB inhibition had no effect on TFPI2-induced HIF-1α upregulation (Supplemental Figure 5, C and D). Given that NF-κB P65 is an important transcription factor that functions in multiple biological activities (38), we aimed to investigate the role of P65 in transcriptional regulation of the CLOCK-BMAL1 complex in GSCs. Analysis of the JASPAR database demonstrated that putative *P65*-binding elements were aligned with the promoters of *CLOCK* and *BMAL1* (Supplemental Figure 5, E–G). ChIP-PCR on GSC272 confirmed that *P65* was bound to *CLOCK* and *BMAL1* promoters (Figure 5, G and H). RT-qPCR results in GSC17 and GSC23 showed that TFPI2 overexpression–induced upregulation of *CLOCK* and *BMAL1* were abolished by pharmacologic inhibition of HIF-1α (Figure 5, I and J) or NF-κB (Figure 5, K and L). Similarly, the upregulated protein levels of CLOCK and BMAL1 in TFPI2-overexpressing GSC17 and GSC23 were negated by the treatment with ACF (Figure 5, M and N) or SC75741 (Figure 5, O and P). In TCGA GBM patient tumors, *CLOCK* was correlated positively with *HIF-1A* and *NFKB1* (Supplemental Figure 5, H and I). Together, these findings indicate that TFPI2 regulates the CLOCK-BMAL1 complex in GSCs through the HIF-1α–NF-κB signaling axis.

### HIF-1α–NF-κB signaling axis mediates TFPI2-induced GSC self renewal.

**Figure 6:**
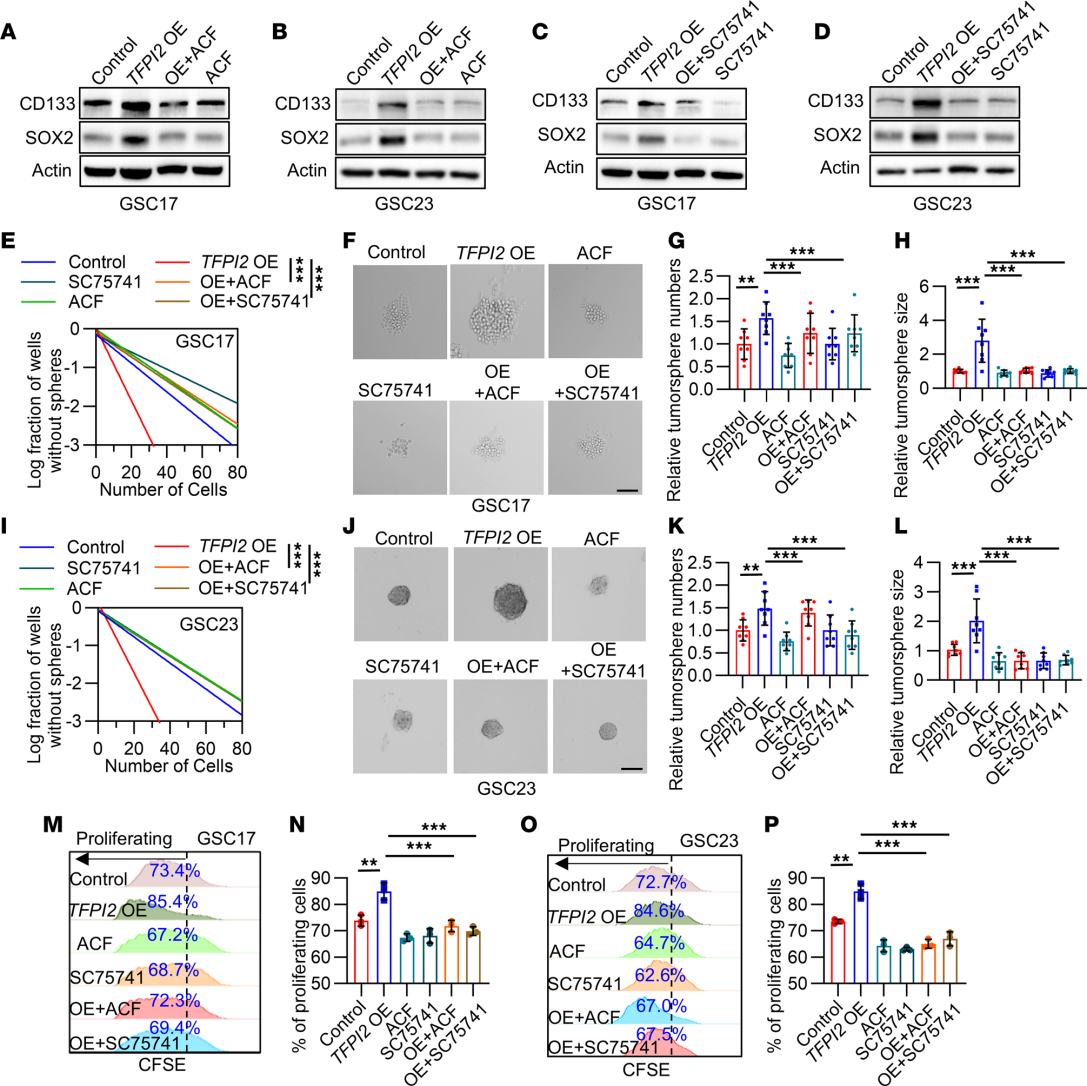
TFPI2-induced GSC self renewal is regulated by the HIF-1α–NF-κB signaling axis. (**A** and **B**) Immunoblots for CD133 and SOX2 in cell lysates of GSC17 (**A**) and GSC23 (**B**) harboring control or *TFPI2* overexpression (OE) and treated with or without HIF-1α inhibitor ACF (2 μM). (**C** and **D**) Immunoblots for CD133 and SOX2 in cell lysates of control and *TFPI2*-OE GSC17 (**C**) and GSC23 (**D**) treated with or without P65 inhibitor SC75741 (5 μM). (**E**) In vitro limiting dilution assays in control and *TFPI2*-OE GSC17 treated with or without ACF (2 μM) or SC75741 (5 μM). (**F**–**H**) Representative images (**F**) and quantification of relative tumorsphere number (**G**) and size (**H**) of control and *TFPI2*-OE GSC17 treated with or without ACF or SC75741. Scale bar: 200 μm. *n* = 8. (**I**) In vitro limiting dilution assays in control and *TFPI2*-OE GSC23 treated with or without ACF (2 μM) or SC75741 (5 μM). (**J**–**L**) Representative images (**J**) and quantification of relative tumorsphere number (**K**) and size (**L**) of control and *TFPI2*-OE GSC23 treated with or without ACF or SC75741. Scale bar: 200 μm. *n* = 8. (**M**–**P**) Representative and quantification of proliferation in GSC17 (**M** and **N**) and GSC23 (**O** and **P**) harboring control or *TFPI2* OE treated with or without ACF (2 μM) or SC75741 (5 μM). *n* = 3. Data from multiple replicates are presented as mean ± SD. **P < 0.01, ***P < 0.001, 1-way ANOVA test (**G**, **H**, **K**, **L**, **N**, and **P**), 2-way ANOVA test (**E** and **I**).

Given that TFPI2 is essential for GSC stemness maintenance (16) and can regulate the HIF-1α–NF-κB–CLOCK–BMAL1 signaling axis in GSCs, we investigated whether this signaling pathway can mediate TFPI2 overexpression-induced GSC self renewal. Pharmacologic inhibition of HIF-1α with its inhibitor ACF or NF-κB with its inhibitor SC75741 negated the upregulation of CD133 and SOX2 at both mRNA and protein levels induced by TFPI2 overexpression in GSC17 and GSC23 (Figure 6, A–D, and Supplemental Figure 6, A and B). Moreover, the treatment with ACF or SC75741 abolished the self renewal (Figure 6, E–L), proliferation (Figure 6, M–P), and apoptotic (Supplemental Figure 6, C–F) effects of overexpressing TFPI2 in GSC17 and GSC23. Together, these results suggest that the HIF-1α–NF-κB signaling axis plays a key role in regulating TFPI2-induced upregulation of GSC self renewal and proliferation, and downregulation of apoptosis.

### Targeting downstream pathways of the TFPI2-CLOCK interplay reduces GSC stemness, activates antitumor immunity, and synergizes with anti-PD1 therapy.

**Figure 7:**
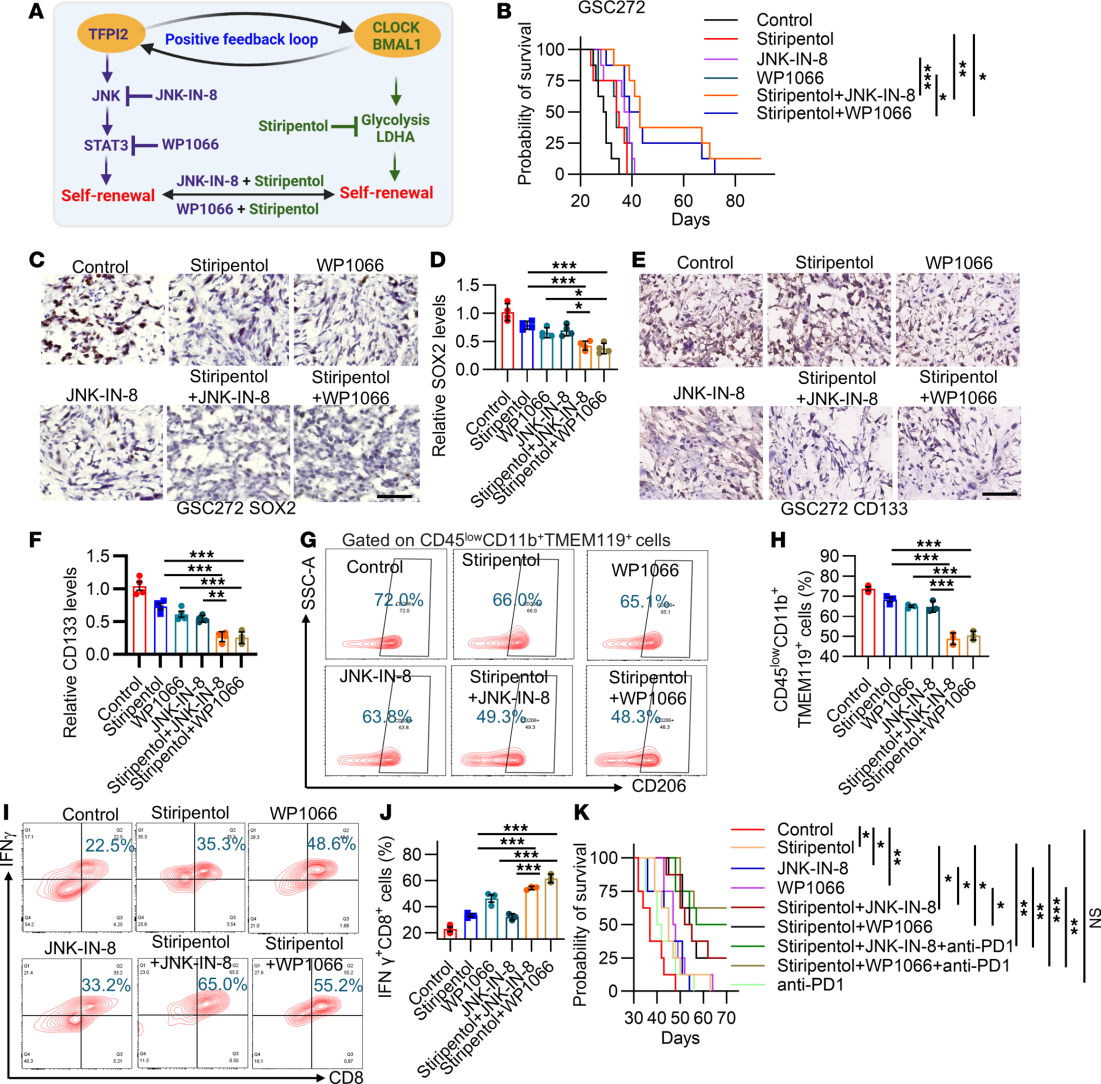
Inhibition of the downstream pathways of the TFPI2-CLOCK signaling loop reduces GSC stemness and immunosuppressive microglia, activates antitumor immunity, and synergizes with anti-PD1 therapy. (**A**) Schematic diagram depicting the positive feedback loop between TFPI2 and the CLOCK-BMAL1 complex in GSCs and the strategy of dual targeting their downstream signaling pathways to block GSC self renewal. (**B**) Survival curves of nude mice implanted with 2 × 10^5^ GSC272 and treated with LDHA inhibitor Stiripentol (150 mg/kg, i.p., every other day), JNK inhibitor JNK-IN-8 (30 mg/kg, i.p., daily), STAT3 inhibitor WP1066 (30 mg/kg, i.p., daily), or Stiripentol in combination with JNK-IN-8 or WP1066 for 2 weeks beginning at day 7. *n* = 8 mice per group. (**C**–**F**) Representative images and quantification of IHC staining for SOX2 (**C** and **D**) and CD133 (**E** and **F**) in GSC272 tumors from mice with indicated treatments. Scale bar: 100 μm. *n* = 4. (**G** and **H**) Representative images (**G**) and quantification (**H**) of flow cytometry for the percentage of CD45^low^CD11b^+^TMEM119^+^CD206^+^ microglia (out of CD45^low^CD11b^+^TMEM119^+^ microglia) in tumors from 005 GSC-bearing mice with indicated treatments. *n* = 3. (**I** and **J**) Representative images (**I**) and quantification (**J**) of flow cytometry for the percentage of CD45^+^CD3^+^CD8^+^IFNγ^+^ T cells (out of CD45^+^CD3^+^CD8^+^ T cells) in tumors from 005 GSC-bearing mice with indicated treatments. *n* = 3. (**K**) Survival curves of C57BL/6J mice implanted with 2 × 10^5^ 005 GSCs and treated with Stiripentol, JNK-IN-8, or WP1066, and Stiripentol in combination with JNK-IN-8 or WP1066 for 2 weeks beginning at day 7, and with anti-PD1 (10 mg/kg, i.p.) treatment on days 11, 14, and 17. *n* = 8 mice per group. Data from multiple replicates are presented as mean ± SD. *P < 0.05, **P < 0.01, ***P < 0.001, 1-way ANOVA test (**D**, **F**, **H**, and **J**), log-rank test (**B** and **K**).

Given the critical role of the CLOCK-TFPI2 interplay in maintaining GSC stemness maintenance and promoting GBM progression, targeting either CLOCK or TFPI2 presents a promising therapeutic strategy for GBM. However, it is unfortunate that no inhibitors are currently available to target either CLOCK or TFPI2. Recently, we and others have demonstrated that activation of glycolysis enzymes, such as LDHA, is required for CLOCK-induced GSC self renewal (20, 26), and inhibition of LDHA with a FDA-approved drug for treating brain disorder Dravet syndrome (39, 40), Stiripentol, can inhibit GBM progression (41). Moreover, we have revealed that TFPI2 can activate the JNK-STAT3 signaling pathway to promote GSC self renewal and GBM progression (16). Therefore, we designed a therapeutic strategy with combined inhibition of LDHA (using LDHA inhibitor Stiripentol) and the JNK-STAT3 signaling axis (using JNK inhibitor JNK-IN-8 or STAT3 inhibitor WP1066) to target the CLOCK-TFPI2 interplay (Figure 7A). We found that the single treatment with Stiripentol, JNK-IN-8, or WP1066 extended the survival in GSC272 PDX model, while the combination therapy with Stiripentol and JNK-IN-8, or Stiripentol and WP1066 resulted in further survival extension, with 25%–37.5% of tumor-bearing mice cleared their tumors after the therapy (Figure 7B). On the histological level, the combination therapies significantly reduced GSC stemness markers SOX2 and CD133 compared with the single treatment (Figure 7, C–F). Our previous studies have shown that CLOCK in GSCs plays a crucial role in promoting the infiltration of immunosuppressive microglia and influencing T cell activation in the GBM TME (16, 21). To confirm the potential synergistic effect of dual inhibition of LDHA and the JNK-STAT3 signaling axis in regulating antitumor immunity, we developed an immunocompetent mouse model by intracranial injection of 005 GSCs. The results demonstrated that the combination therapy with Stiripentol and JNK-IN-8, or Stiripentol and WP1066 significantly reduced CD45^low^CD11b^+^TMEM119^+^CD206^+^ immunosuppressive microglia (Figure 7, G and H) and upregulated CD45^+^CD3^+^CD8^+^IFNγ^+^– or CD45^+^CD3^+^CD8^+^CD69^+^–activated CD8^+^ T cells (Figure 7, I and J, and Supplemental Figure 7, A and B) when compared with the single treatment. However, CD45^+^CD3^+^CD4^+^IFNγ^+^– or CD45^+^CD3^+^CD4^+^CD69^+^–activated CD4^+^ T cells were not affected (Supplemental Figure 7, C–F). Survival studies showed that the combination therapy led to complete tumor clearance in 25% of tumor-bearing mice, with the rate increasing to 50%–62.5% when further combined with anti-PD1 therapy (Figure 7K). The triple therapies (Stiripentol + JNK-IN-8 + anti-PD1 and Stiripentol + WP1066 + anti-PD1) activated T cell memory, as all cured mice remained tumor free when rechallenged with 005 GSCs (Supplemental Figure 7G). Together, these findings suggest that the combination therapy targeting the downstream signals of CLOCK and TFPI2 is a promising therapeutic strategy for GBM.

## Discussion

Our recent studies have demonstrated that *CLOCK* and *TFPI2* are amplified in approximately 4%–6% of GBM cases (16, 20), suggesting that targeting either factor or its downstream signaling pathways may provide therapeutic benefit to this molecularly defined subset of patients. In this study, we uncovered a ‘symbiotic exclusivity’ pattern between *CLOCK* and *TFPI2* in patients with GBM, inducing a positive feedback loop between them to promote GSC self renewal, microglia immunosuppression, and GBM progression. Mechanistically, the CLOCK-BMAL1 complex transcriptionally upregulates TFPI2 expression in GSCs, and, reciprocally, TFPI2 enhances the expression of the CLOCK-BMAL1 complex through activating the HIF-1α–NF-κB signaling pathways. Therefore, the results of the current study highlight that therapies targeting CLOCK or TFPI2 could expand the pool of potential treatment responders, particularly for patients harboring high expression of either factor. In the GBM PDX GSC272 model, dual inhibition of LDHA, downstream of CLOCK, and the JNK-STAT3 axis, downstream of TFPI2, demonstrated a synergistic effect, completely eradicating tumors in a subset of GBM-bearing mice. Similar results were observed in immunocompetent 005 GSC model and the antitumor effects were further heightened when the treatment strategy was combined with anti-PD1 immunotherapy. Together, our findings uncover a regulatory circuit underlying GSC stemness maintenance and its connection with tumor immunity and underscore the therapeutic potential of targeting the TFPI2-CLOCK interplay in GBM. Moreover, our results provide a conceptual and translational basis for exploring ‘symbiotic exclusivity’ as a targetable vulnerability in a broad spectrum of cancers.

TFPI2 is a Kunitz-type serine protease inhibitor capable of inhibiting a broad spectrum of proteases (42). We recently demonstrated that TFPI2 is highly expressed in GBM tumors, although it is amplified in only 4%–6% of cases (16), suggesting that its expression may be regulated by other factors. In this study, we revealed the molecular mechanism showing that the CLOCK-BMAL1 complex can directly and transcriptionally upregulate TFPI2 expression in GSCs. In addition, we observed similar expression and amplification patterns of CLOCK in GBM (20). The results of this study observed in GSCs are consistent with previous studies showing that TFPI2 can regulate the NF-κB pathway in liver sinusoidal endothelial cells (43) and macrophages (44). As a result, the P65 of activated NF-κB binds to the promoters of CLOCK and BMAL1, transcriptionally upregulating the CLOCK-BMAL1 complex in GSCs. In exploring the molecular link between TFPI2 and NF-κB signaling, we discovered that TFPI2 upregulates HIF-1α to activate NF-κB, aligning with previous work showing a HIF-1α–NF-κB connection in GSCs during the development of radioresistance (45). The interaction between the CLOCK-BMAL1 complex and TFPI2 establishes a positive feedback loop that sustains GSC self renewal and drives GBM progression. These findings not only help explain the high expression of CLOCK and TFPI2 in GBM tumors despite their low amplification rates, but also highlight their potential as therapeutic targets in GBM.

Despite the efforts to develop targeted therapies, including those targeting the RTK-RAS-PI3K-PTEN pathway, these approaches have failed to significantly improve patient outcomes due to the development of resistance (46–48). Therapeutic resistance in GBM is largely attributed to tumor heterogeneity and the presence of GSCs (13, 49). GSC populations play an important role in GBM initiation and resistance to various treatments (49, 50). Therefore, identifying key regulators responsible for GSC maintenance has become an area of growing focus in recent years (51). With respect to GSC biology, we and others have demonstrated that the CLOCK-BMAL1 complex and TFPI2 are critical for GSC self renewal by regulating glycolysis enzyme LDHA and the JNK-STAT3 signaling axis, respectively (16, 20, 26, 52). Depletion of the CLOCK-BMAL1 complex and TFPI2 in GSCs reduces tumor progression, extends survival, and synergizes with immunotherapy in GBM mouse models (16, 20, 21, 26, 52), suggesting the translational potential of targeting the CLOCK-BMAL1 complex and TFPI2. Given the lack of direct and specific inhibitors against the CLOCK-BMAL1 complex or TFPI2, this study designed a therapeutic strategy by simultaneously targeting their key downstream effectors, LDHA and the JNK-STAT3 signaling axis. Our findings demonstrate a synergistic antitumor effect from dual inhibition of LDHA and the JNK–STAT3 signaling axis, supporting the enhanced translational potential of this combination therapy for GBM patients.

Emerging evidence reveals that symbiotic interactions between tumor cells and immune cells are critical for tumor progression and immunosuppression (53–59). With respect to GBM, CLOCK- and TFPI2-regulated GSCs are connected with immunosuppression via regulating microglia infiltration and immunosuppressive polarization (16, 20, 21, 52). GBM is considered as a ‘cold’ tumor, marked by a paucity of T cells and abundant infiltration of immunosuppressive microglia and macrophages (16, 21, 41, 48, 53–58, 60–63). In this study, we found that blockade of the TFPI2-CLOCK interplay by dual inhibition of LDHA and JNK-STAT3 axis reduced immunosuppressive microglia and promoted the activation of T cells, remodeling the TME from “cold” to “hot”. Our preclinical trial demonstrated that the dual treatment strategy synergized with anti-PD1 therapy, leading to durable antitumor responses and complete tumor regression in 50%–62.5% of GBM tumor-bearing mice. Together, our mechanistic insights into GSC stemness regulation and antitumor responses in GBM models support the rationale for clinical trials to assess the efficacy and safety of combination therapies in GBM patients.

## Methods

### Sex as a biological variable.

Female mice were used in this study. There are no reported sex differences among patients with GBM with high and low levels of CLOCK and TFPI2. Sex was not considered as a biological variable in this study.

### Mice and intracranial xenograft tumor models.

Female C57BL/6J mice (#000664) and nude mice (#007850) at 5–6 weeks of age were purchased from the Jackson Laboratory. Animals were kept in rooms with controlled temperature (21–23°C) and humidity (30%–70%) and maintained on a 12-hour light/dark cycle. All animal experiments were conducted with approval from the Institutional Animal Care and Use Committee (IACUC). The intracranial xenograft tumor models were generated as we described previously (16, 41, 64). In brief, mice were anesthetized by isoflurane through the IMPAC6 Anesthesia System. A small hole was drilled into the skull of mice using the dental drill, positioned 1.2 mm anterior and 3.0 mm lateral to the bregma. Mice were placed and stabilized into the stereotactic apparatus. 5 μL of GSCs in FBS-free culture medium were slowly injected into the right caudate nucleus 3 mm below the surface of the brain using a 10 μL Hamilton syringe with an unbeveled 30-gauge needle. The incision was closed with Vetbond glue and pain relief was provided with three subcutaneous doses of meloxicam (5 mg/kg). On day 7 post intracranial injection, mice were treated with SR9009 (Selleck Chemicals, #S8692, 100 mg/kg/day, i.p.), WP1066 (MCE, #HY-15312, 30 mg/kg/day, i.p.), JNK-IN-8 (MCE, # HY-13319, 30 mg/kg/day, i.p.), Stiripentol (MCE, #HY-103392, 150 mg/kg/day, i.p.). Anti-PD1 (10 mg/kg, i.p.) was given on day 11, 14, and 17 post-orthotopic injection. Mice with neurological deficits or moribund appearance were sacrificed. At the end of the experiment, the brains of tumor-bearing mice were isolated following with or without transcranial perfusion with 4% paraformaldehyde (PFA, ThermoFisher, #J61899.AK) for cryosectioning or tumor-derived immune cell isolation for following analysis.

### Cell culture.

CT2A cells were purchased from the American Type Culture Collection (ATCC), and cultured in Dulbecco’s Modified Eagle Medium (Gibco, #11995-065) containing 10% fetal bovine serum (FBS, Fisher Scientific, #16140071) and 1:100 antibiotic-antimycotic (Gibco, #15140-122). Patient-derived GSCs (GSC272, GSC17 and GSC23) were gifted by Dr. Frederick F. Lang from the Brain Tumor Center (The University of Texas MD Anderson Cancer Center). Mouse GBM tumor-derived 005 GSCs and QPP7 GSCs were given by Dr. Samuel D. Rabkin (Massachusetts General Hospital) and Dr. Jian Hu (The University of Texas MD Anderson Cancer Center), respectively. For stemness maintenance, human and mouse GSCs, and CT2A cells were cultured in neural stem cell proliferation media (Millipore, #SCM005) containing 20 ng/mL basic fibroblast growth factor (PeproTech, #100-18B) and epidermal growth factor (PeproTech, #AF-100-15). All cells were verified to be free of mycoplasma and were kept at 37°C with 5% CO2. Cells were treated with SC75741 (MCE, #HY-10496), Acriflavine (ACF, Sigma, #A8126) and SR9009 (Selleck Chemicals, #S8692) for 8 hrs for mRNA expression analysis and 24 hrs for protein expression analysis.

### Plasmids and viral transfections.

Short hairpin RNA (shRNA) targeting human *TFPI2*, *CLOCK* and *BMAL1* and mouse *Tfpi2*, *Clock* and *Bmal1* in the pLKO.1 vector (Sigma, #SHC001) were used in this study. Lentiviral particles were produced as we described previously (16, 41, 62). Briefly, 8 μg shRNA plasmid, 4 μg psPAX2 plasmid (Addgene, #12260), and 2 μg pMD2.G plasmid (Addgene, #12259) were transfected into 293T cells in 100-mm dishes using Lipofectamine 2000 (Invitrogen, #13778150). Then the viral supernatant was collected 48 and 72 hrs after transfection and filtered through a 0.45 μm filter. Cells were infected with lentiviral particles containing 10 μg/mL polybrene (Millipore, #TR-1003-G). Transfected cells were then selected using puromycin (10 μg/mL; Millipore, #540411) after 48 hrs and tested for protein expression by western blot. The following human and mouse shRNA sequences (*TFPI2*: #1: TRCN0000373822 and #2: TRCN0000072725; *CLOCK*: #1: TRCN0000018974 and #2: TRCN0000018975; *BMAL1*: #96: TRCN0000019096 and #98: TRCN0000019098; *OLFML3*: #1: TRCN0000186745 and #3: TRCN0000203502; *Tfpi2*: #1: TRCN0000271824 and #2: TRCN0000271717; *Clock*: #1: TRCN0000095686 and #2: TRCN0000306474; and *Bmal1*: #54: TRCN0000095054 and #57: TRCN0000095057) were selected for further use following the validation. Doxycycline-inducible plasmids were generated by cloning the desired shRNA sequence (sh*CLOCK* #2) into a pLKO.1 vector using the Gateway Cloning System (Thermo Fisher Scientific). After transfection, cells were treated with doxycycline (2 μg/mL) for 48 hrs to induce CLOCK knockdown.

For overexpression, GSCs were transfected with a *TFPI2* and CLOCK overexpression plasmid generated through a standard cloning strategy as previously described (16, 20). Plasmids of human Tagged ORF Clone of *CLOCK* (Origene, # RC221408), and *TFPI2* (Origene, #RC202760) and mouse Tagged ORF Clone of *Clock* (Origene, #MR226315), and *Tfpi2* (Origene, #MR202782) were used. Briefly, the gene open reading frame (Myc-DDK-tagged) was excised from a pCMV6-Entry vector (Origene, #RC202760) and subcloned into the pLenti-C-mGFP lentiviral expression vector (Origene, #PS100071). Both plasmids were digested with the restriction enzymes MluI (Promega, #R6381) and SgfI (Promega, #R7103) in the presence of Restriction Digest Buffer C to maximize digestion efficiency. Post-digestion, the DNA was treated with Antarctic Phosphatase for 3 hrs to prevent vector self-ligation. The digested insert was ligated into the linearized pLenti-C-mGFP vector using T4 DNA ligase (Thermo Scientific, #B69). The ligation mixture was transformed into high-efficiency chemically competent *Escherichia coli* cells (Thermo Scientific, #C737303) and cultured in Lysogeny Broth (LB; Fisher BioReagents, #BP9723) for plasmid propagation. After recovery, transformed cells were plated on LB agar containing 34 μg/mL chloramphenicol (Fisher BioReagents, #BP904) and incubated at 37°C for 16 hrs to select for clones containing the gene expression vectors. Selected colonies were picked and inoculated in LB broth supplemented with 34 μg/mL chloramphenicol to maintain selection. Plasmid DNA was purified using the QIAprep Spin Miniprep Kit (Qiagen, #27106) and subsequently transfected into cells using a lentiviral transfection protocol as previously described (16, 20, 64).

### Immunoblotting.

Immunoblotting was performed following standard protocol. Briefly, cells were lysed with RIPA buffer (Thermo Fisher Scientific, #89901) supplemented with protease inhibitor cocktail (Millipore, #11697498001) and placed on ice for 10 min. The concentration of the protein was measured using the BCA Protein Assay Kit (Thermo Fisher Scientific, #PI23225). The cell lysate was mixed with 4x Laemmli sample buffer and then boiled at 95°C for 10 min. Then the boiled protein sample was added to SurePAGE gels (GenScript, #M00653). The gels were run at a constant voltage of 60 V and then transferred to 0.2 μm nitrocellulose (NC) membrane (Bio-Rad, #1620112) using standard protocol for 30 min in the Trans-Blot Turbo system (Bio-Rad). NC membranes were blocked using 5% non-fat dry milk in TBST for 1 hr at room temperature and were then incubated with primary antibodies (1:1000 dilution) overnight at 4°C. Membranes were then washed 3 times in TBST and were then incubated with HRP-conjugated secondary antibodies (1:1000 dilution; CST, #7076S and #7074S) for 1 hr at room temperature. Washed three times, membranes were incubated with ECL substrate and then imaged using a ChemiDoc Imaging System (Bio-Rad). Antibodies were purchased from the indicated companies, which include β-actin (Cell Signaling Technology, #3700S), TFPI2 (Abcam, #ab186747), CLOCK (Cell Signaling Technology, #5157S), BMAL1 (Cell Signaling Technology, #14020), OLFML3 (Abcam, #ab111712), P-P65 (Cell Signaling Technology, #3033S), P65 (Cell Signaling Technology, #8242S), HIF-1α (Cell Signaling Technology, #14179), CD133 (Biosis, #BS-4770R) and SOX2 (Abcam, #ab97959).

### RT-qPCR.

Cells were pelleted and RNA of cells was isolated by the RNeasy Mini Kit (Qiagen, #74106) as described previously. The concentration of RNA was measured using the NanoDrop spectrophotometers. Then RNA was reverse transcribed into cDNA using the All-In-One 5X RT MasterMix (Applied Biological Materials, #G592) in T100 Thermal Cycler (Bio-Rad). RT-qPCR was performed using SYBR Green PCR Master Mix (Bio-Rad, #1725275) in CFX Connect Real-Time PCR Detection System (Bio-Rad). Detailed information about the primers used was listed in Supplemental Table 1. The expression of each gene was normalized to the housekeeping gene human *GAPDH* and mouse *Actin*.

### ChIP-PCR.

GSE134974, which includes BMAL1 chromatin immunoprecipitation sequencing (ChIP-seq) data from patient-derived GSCs and normal human NSCs, was utilized in this study, The ChIP-seq data were analyzed using the Integrative Genomics Viewer (Broad Institute). ChIP-PCR was performed using the commercial PierceTM Magnetic ChIP kit (ThermoFisher, #26157) as previously described. In brief, GSC272 cells were cross-linked by 1% PFA for 10 min and then reactions were quenched by glycine for 5 min at room temperature. Cells were incubated with a Membrane Extraction Buffer on ice for 10 min. Then the chromatin fragmentation was produced by Mnase digestion followed by sonication in a procedure of 20-sec pulse at 3-walt power. Solubilized chromatin was next incubated with p65 antibody overnight at 4°C. IP reactions were incubated with ChIP Grade Protein A/G Magnetic Beads for 2 hrs at 4°C. Immune complexes were then washed with IP wash buffer I and IP wash buffer II. Elution was conducted by adding elution buffer at 65°C for 30 min, and then reverse cross-linking was performed using proteinase K (20 mg/mL) and NaCl (5M) at 65°C for 1.5 hrs. Eluted DNA was purified using DNA Clean-up Column and for following qPCR experiments. The CLOCK primers were designed according to the E-box of the human CLOCK gene and were also listed in Supplemental Table 1.

### IHC and IF.

IHC and IF were performed following an established protocol as previously described (20, 64). Briefly, mouse brains were transferred from 4% PFA to a 50 mL Falcon tube containing 15% sucrose in PBS with 0.01% sodium azide and incubated for 48 hrs at 4°C. Then the brains were then transferred to 30% sucrose in PBS for an additional 48 hrs for further preservation. The brains were fully embedded in the OCT compound and frozen at -80°C for long-term storage. Cryosectioning was performed using a Leica CM1860 UV Cryostat, and the brains were sliced into 10 μm sections for downstream analysis. For IHC staining, the slides were first placed in room temperature for 30 min after taken out from refrigerator, then washed three times with PBS and fixed in 4% PFA for 30 min. To permeabilize the cell membrane, the sections were incubated in 0.3% Triton X-100 in PBS for 10 min at room temperature. Next, the slides were washed three times and blocked with 10% goat serum for 1 hr to reduce non-specific binding. The slides were then incubated overnight at 4°C with primary antibodies diluted at 1:200 in a suitable antibody dilution buffer. Then, the slides were incubated with relevant secondary antibodies (Cell Signaling Technology, #7076S and #7074S) for 1 hr at room temperature. After another round of PBS washes, the staining was developed using DAB Quanto (Epredia, #TA125QHDX) to produce a brown chromogenic signal at the site of antibody binding. The nuclei were counterstained with hematoxylin to provide contrast and to facilitate cellular localization of the staining signal. Images were captured using the Aperio AT2 whole slide scanner under consistent imaging conditions to ensure reproducibility and treated using software ImangeScope X64. Positive staining signals were quantified using ImageJ software (NIH, Bethesda, MD) with the IHC Profiler plug-in to objectively assess the staining intensity and distribution. The following primary antibodies were used for IHC: CD133 (Biosis, #BS-4770R) and SOX2 (Abcam, #ab97959). For immunofluorescence, a standard protocol. In brief, slides of cryosection were left at room temperature for 30 min, then fixed in 10% PFA for 30 min before permeabilization. To permeabilize the cell membrane, 0.3% (v/v) Triton X-100 in PBS was applied for 30 min at room temperature. Antigen retrieval was performed by boiling the sections four times in sodium citrate buffer (0.01 M, pH 6) using a microwave. Then slides were washed with PBS 3 times and blocked with 5% goat serum for 30 min. Primary antibody was added to the specimen for 1 hr at room temperature and then overnight at 4°C. Then the slides were washed with PBS 3 times to remove the unbound primary antibodies. Corresponding secondary antibody cocktails were prepared and added to the specimen for 1 hr at room temperature. Cell nuclei were stained with 4′,6-diamidino-2-phenylindole/anti-fade mounting medium (Vector Laboratories, #H-1200-10). Immunofluorescence images were captured using the Nikon AX/AX R Confocal Microscope System. ImageJ software was used to quantify the relative intensity of the stained protein.

### Flow cytometry.

For intratumoral immune cell analysis, the cell isolation and staining were performed following an established protocol as previously described (16). In brief, mouse brains were collected after transcardiac perfusion and homogenized on ice using glass cell tissue homogenizer with pre-cold Hank’s Balanced Salt Solution (HBSS). Cells were then pelleted by centrifuge and resuspended in 30% Percoll (GE Healthcare, #17-0891-01). Next, cells in 30% Percoll were gently laid on top of the 70% Percoll. After centrifuging at 1,200 g for 30 min at 4 °C with accelerator 7 and breaker 0, the top layer of myelin and debris were removed, and the interphase of immune cells were collected and then resuspended in PBS for further analysis. For cell labeling, the number-matched tumor single-cell suspensions were incubated with the TruStain FcX (anti-mouse CD16/32) Antibody (BioLegend, #103132) and True-Stain Monocyte Blocker (BioLegend, #426102) for 30 min. After washing, cells were incubated with a cocktail of antibodies. For microglia staining, Percp/Cy5.5 anti-mouse CD45 (BioLegend, #103132), PE/Cy7 anti-mouse/human CD11b (BioLegend, #101216), AF488 anti-mouse TMEM119 (Invitrogen, # 53-6119-82), and AF647 anti-mouse CD206 (BD Bioscience, #565250) were used. For T cell staining, the antibody cocktail contained Percp/Cy5.5 anti-mouse CD45 (BioLegend, #103132), AF488 anti-mouse CD3 (BioLegend, #100210), BUV395 anti-mouse CD4 (BD Bioscience, #740208), BV711 anti-mouse CD8 (BioLegend, #100747), PE/Cy7 anti-mouse CD69 (BioLegend, #104512), and APC/Cy7 anti-mouse IFN-γ (BioLegend, #505850). After washing with FACS buffer, cells were incubated with fixation buffer (BioLegend, #420801). Then the labeled cells were analyzed using BD FACSymphony flow cytometer and FlowJo v10.8.1 software.

### Proliferation analysis.

Cell proliferation was measured using the CellTrace carboxyfluorescein succinimidyl ester (CFSE) Cell Proliferation Kit (Invitrogen, #C34554). In brief, 10^6^ GSCs were collected and incubated with CFSE working solution (1:1000) for 20 min at 37°C. Then complete cell culture medium was added to stop the staining. Then cells were washed and cultured in the dark incubator for 5 days and were harvested for further flow cytometry analysis. The percentage of CFSE-positive peaks was analyzed by FlowJo v10.8.1 software.

### Apoptosis analysis.

Cell apoptosis was measured using Apotracker Green (BioLegend, #427402) as described previously (16). In brief, 10^5^ GSCs were collected and stained with Apotracker (1:10 dilution). Then 5 μL propidium iodide (PI) solution (BioLegend, # 421301) was added to label the necrotic and late apoptotic cells. Cells were washed with PBS three times. Fluorescein isothiocyanate (FITC) and propidium iodide signals were chosen and analyzed in the BD FACSymphony flow cytometer.

### Tumorsphere formation assay.

Cells were plated in 96-well plates at densities ranging from 0 to 80 GSCs per well, with eight technical biological replicates for each condition. Cells were maintained for 10 days before evaluating sphere formation. Spheres with a diameter greater than 10 cells were included in the analysis while wells less than that were recorded as negative. Statistical analysis was performed using the extreme limiting dilution assay (ELDA) online tool (65). Tumorspheres (seeded with 80 GSCs) counted and single tumorsphere (seeded with 5 GSCs) in each group was imaged.

### Computational analysis of human GBM datasets.

For analyzing the gene alterations of human GBM, we downloaded the copy-number data from TCGA datasets or other available sources via cBioPortal and analyzed them as previously described (16). The microarray data and RNA-seq data (GSE140409 and GSE134974) were downloaded from public GEO datasets. The DEGs were screened out with fold change (FC) ≥ 2. GSEA on hallmark pathways (shC versus sh*TFPI2* from the GSE232486 dataset) were performed using GSEA software 4.1.0. The scRNA-seq analyses were performed using the Talk2Data platform (BioTuring) and the Broad Institute Single-Cell Portal. scRNA-seq data of EGAS00001004656 was used to analyze the expression pattern of *TFPI2* and *CLOCK* in GSCs and GBM cells. Based on low and high *TFPI2* and *CLOCK* expressions in GSCs, GBM patients were regrouped as low and high subgroups and the correlation between them in GBM patients was conducted using the scRNA-seq data of GSE182109.

### Statistics.

Statistical analysis was carried out using GraphPad Prism 10 (GraphPad Software, USA). Measurement data are presented as means ± SD. Pearson correlation analysis was used to calculate the Pearson correlation coefficient (*R* value) and P value. Survival analysis was performed using the Log rank (Mantel-Cox) test. Comparisons between 2 groups were made with Student’s 2-tailed *t* test, while comparisons among multiple groups were assessed using 1-way ANOVA and 2-way ANOVA followed by Tukey’s post hoc test. Statistical significance was defined as *P* < 0.05.

### Study approval.

All animal experiments were performed with the approval of the IACUCs at Cleveland Clinic.

### Data availability.

The data supporting the findings of this study are available within this article and within the Supporting Data Values file. The human TCGA GBM amplification data is available at cBioPortal. The RNA-seq data of GSC272 cells with shC versus sh*TFPI2* are obtained from the GEO database (GSE232486). External single-cell data used in this study are available in the NCBI’s GEO database (GSE182109) and the European Genome-Phenome Archive (EGAS00001004656).

## Author contributions

FZ, LP, YL, and FK performed the experiments. FZ and PC wrote the manuscript. PC designed and supervised the study. All authors have read and approved the article.

## Conflict of interest

The authors have declared that no conflict of interest exists.

## Funding support

This work is the result of NIH funding, in whole or in part, and is subject to the NIH Public Access Policy. Through acceptance of this federal funding, the NIH has been given a right to make the work publicly available in PubMed Central.

- NIH R01NS124594 (PC).
- NIH R01NS127824 (PC).
- DoD Career Development Award W81XWH-21-1-0380 (PC).
- Cancer Research Institute CLIP Grant CRI13662 (PC).
- VeloSano Award (PC).

## Supplementary Material

Supplemental data

Unedited blot and gel images

Supporting data values

## Version 1 03/17/2026

In-Press Preview

## Version 2 05/15/2026

Electronic publication
