# Supplementary material for: Symbiotic exclusivity between CLOCK and TFPI2 drives stemness and immunosuppression in glioblastoma models
Source: J Clin Invest. 2026 Mar 17;136(10):e199056. doi: 10.1172/JCI199056 (PMC13178667; doi:10.1172/JCI199056)
Supplement: Supplemental data [file jci-136-199056-s051.pdf]

Supplemental Figure 1

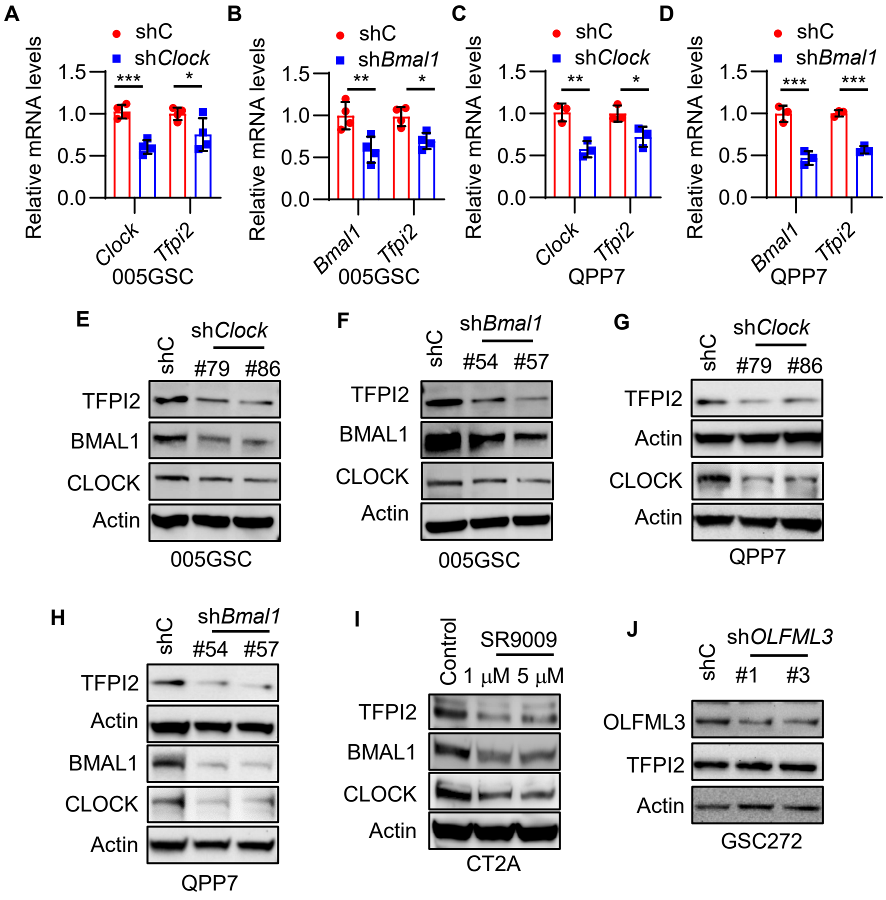

Supplemental Figure 1. TFPI2 is regulated by the CLOCK/BMAL1 complex in GSCs.
(A) RT-qPCR for *Clock* and *Tfpi2* in 005 GSCs harboring shRNA control (shC) and Clock shRNA (sh*Clock*). n = 4.
(B) RT-qPCR for *Bmal1* and *Tfpi2* in 005 GSCs harboring shC and sh*Bmal1*. n = 4.
(C) RT-qPCR for *Clock* and *Tfpi2* in QPP7 GSCs harboring shC and sh*Clock.* n = 3.
(D) RT-qPCR for *Bmal1* and *Tfpi2* in QPP7 GSCs harboring shC and sh*Bmal1*. n = 3.
(E and **F)** Immunoblots for CLOCK, BMAL1, and TFPI2 in 005 GSCs harboring shC, sh*Clock* (**E**), or sh*Bmal1* (**F**).
(G and **H)** Immunoblots for CLOCK, BMAL1, and TFPI2 in QPP7 GSCs harboring shC, sh*Clock* (**G**), or sh*Bmal1* (**H**).
(I) Immunoblots for TFPI2, BMAL1 and CLOCK in CT2A cells treated with or without SR9009 (1 and 5 μmol/L).
(J) Immunoblots for OLFML3 and TFPI2 in GSC272 harboring shC, sh*OLFML3.*

Data from multiple replicates are presented as mean ± SD. *, $P < 0.05$, **, $P < 0.01$, ***, $P < 0.001$, Student's t test (A, B, C and D).

Supplemental Figure 2

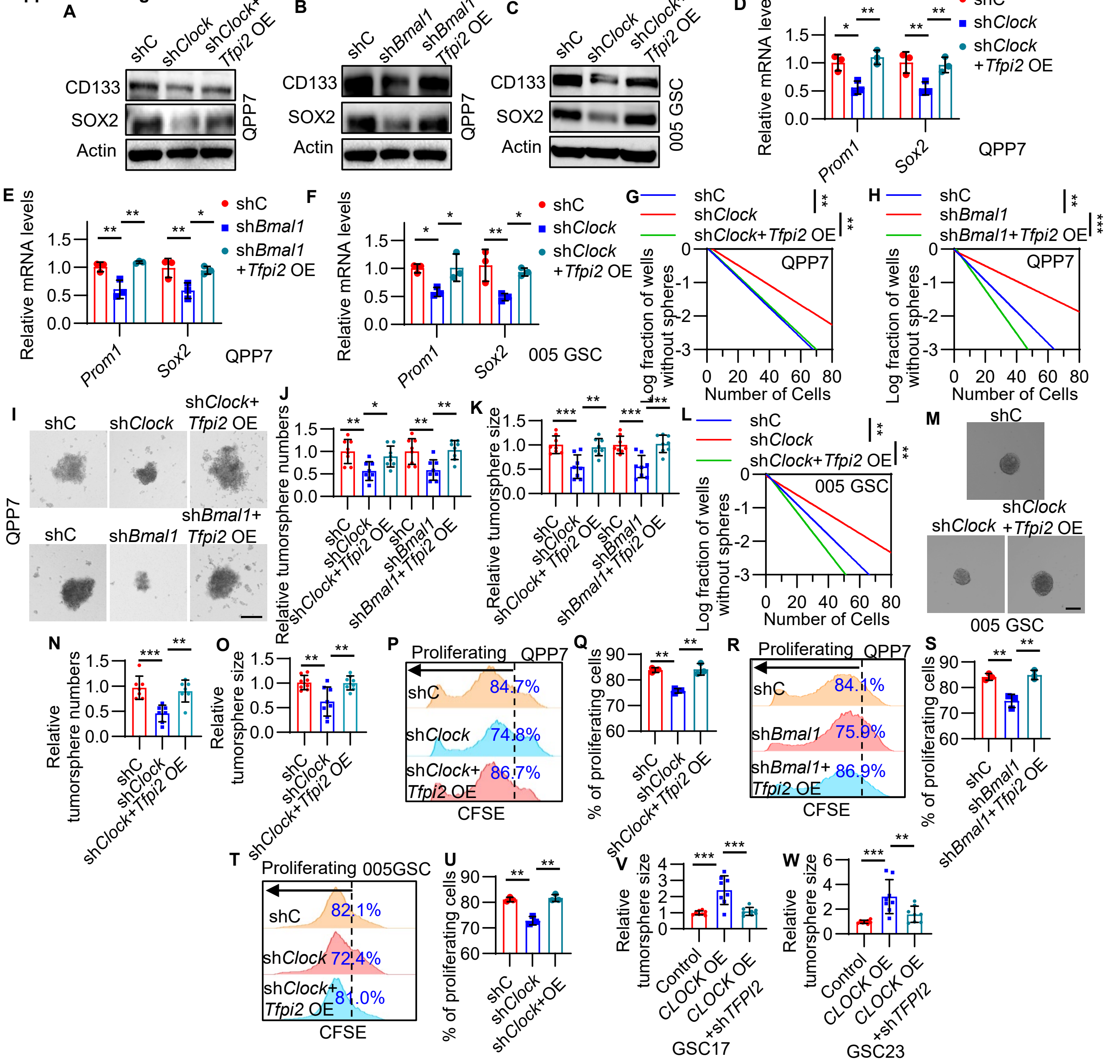

Supplemental Figure 2. CLOCK/BMAL1 depletion-induced impairment of GSC self-renewal is rescued by TFPI2 overexpression.

(A-C) Immunoblots for CD133 and SOX2 in QPP7 GSCs (**A** and **B**) or 005 GSCs (**C**) harboring shRNA control (shC), *Clock* shRNA (sh*Clock*), or sh*Bmal1* with or without *Tfpi2* overexpression (OE).

(D-F) RT-qPCR for *Prom1* and *Sox2* in QPP7 GSCs (**D** and **E**) or 005 GSCs (**F**) harboring shC, sh*Clock*, or sh*Bmal1* with or without *Tfpi2* OE. n = 3.

(G and H) *In vitro* limiting dilution assays in QPP7 GSCs harboring shC, sh*Clock* (**G**) or sh*Bmal1* (**H**) with or without *Tfpi2* OE.

(I-K) Representative images (**I**) and quantification of relative tumorsphere number (**J**) and size (**K**) of QPP7 GSCs harboring shC, sh*Clock* or sh*Bmal1* with or without *Tfpi2* OE. Scale bar, 200 μm. n = 8.

(L) *In vitro* limiting dilution assays in 005 GSCs harboring shC and sh*Clock* with or without *Tfpi2* OE.

(M-O) Representative images (**M**) and quantification of relative tumorsphere number (**N**) and size (**O**) of 005 GSCs harboring shC and sh*Clock* with or without *Tfpi2* OE. Scale bar, 200 μm. n = 7.

(P-U) Representative images and quantification of proliferation of QPP7 GSCs (**P-S**) or 005 GSCs (**T** and **U**) harboring shC, sh*Clock*, or sh*Bmal1* with or without *Tfpi2* OE. n = 3.

(V and W) Quantification of relative tumorsphere size of GSC17 **(V)** and GSC23 **(W)** harboring Control or *CLOCK* OE with or without sh*TFPI2*. n = 8.

Data from multiple replicates are presented as mean ± SD. *, $P < 0.05$, **, $P < 0.01$, ***, $P < 0.001$, one-way ANOVA test (D, E, F, J, K, N, O, Q, S, U, V and W), two-way ANOVA test (G, H and L).

Supplemental Figure 3

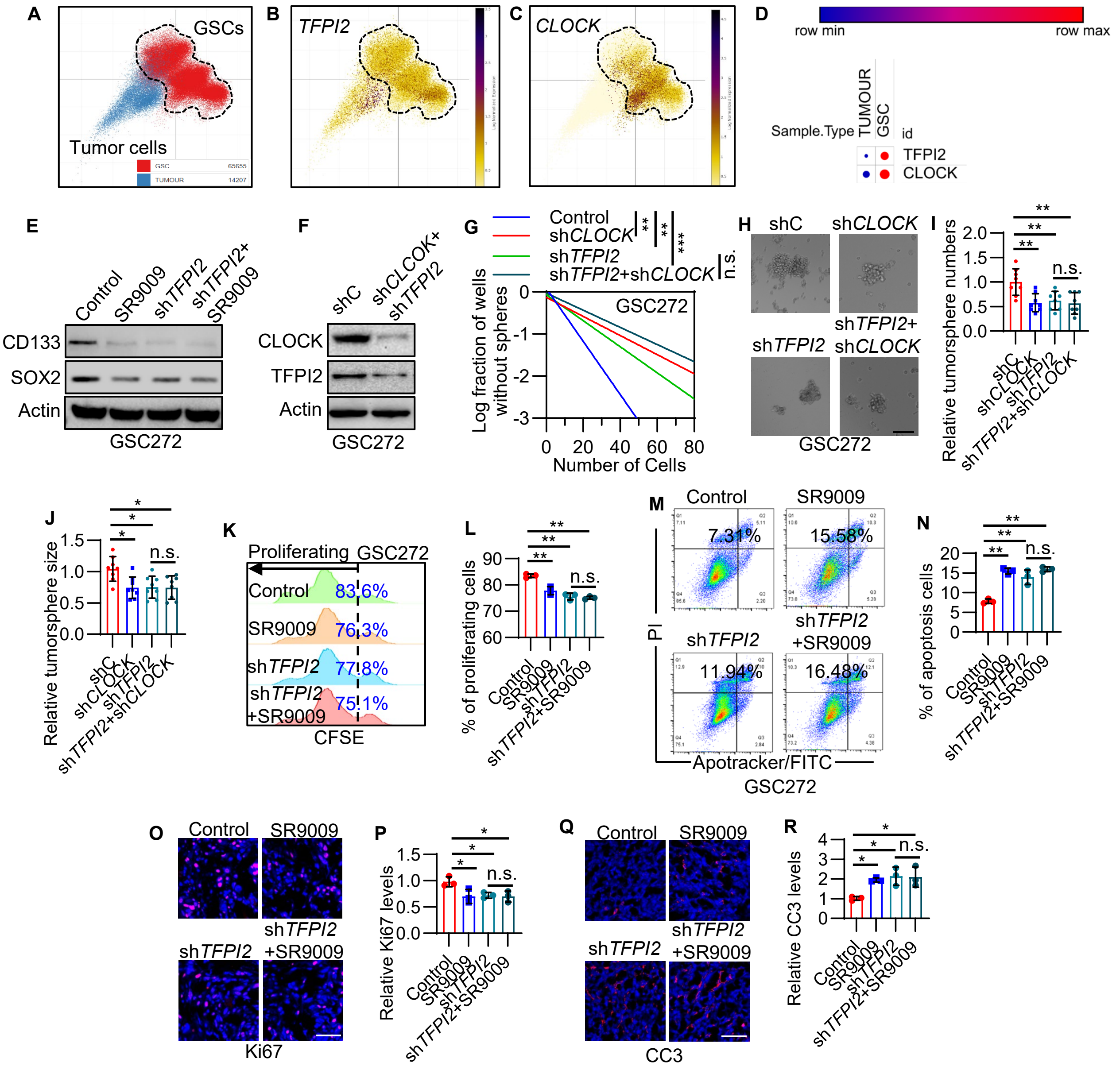

Supplemental Figure 3. Dual inhibition of CLOCK and TFPI2 does not generate additional effect on GSC stemness, proliferation and apoptosis.

(A) High-resolution uniform manifold approximation and projection (UMAP) plots of tumor cells and GSCs in GBM patient tumors. The analysis was based on the scRNA-seq dataset (EGAS00001004656).

(B and **C)** UMAP showing the expression of *TFPI2* (**B**) and *CLOCK* (**C**) in GSC subpopulations. The darker color represents a higher expression.

(D) Dot color intensity represents the average expression of *TFPI2* and *CLOCK* in GSCs and GBM cells.

(E) Immunoblots for CD133 and SOX2 in GSC272 harboring shRNA control (shC) and *TFPI2* shRNA (sh*TFPI2*) treated with or without SR9009 (5 μM).

(F) Immunoblot for TFPI2 and CLOCK in GSC272 harboring shC, and sh*CLOCK* combined with sh*TFPI2* (sh*CLOCK*+sh*TFPI2*).

(G) *In vitro* limiting dilution assays in GSC272 harboring shC, sh*CLOCK*, sh*TFPI2*, and sh*CLOCK*+sh*TFPI2*.

(H-J) Representative images **(H)** and quantification of relative tumorsphere number (**I**) and size (**J**) of GSC272 expressing shC, sh*CLOCK*, sh*TFPI2*, and sh*CLOCK*+ sh*TFPI2*. Scale bar, 200 mm. n = 8.

(K and **L)** Representative (**K**) and quantification (**L**) of proliferation in GSC272 harboring shC and sh*TFPI2* treated with or without SR9009 (5 μM). n = 3.

(M and **N)** Representative (**M**) and quantification (**N**) of apoptosis in GSC272 harboring shC and sh*TFPI2* treated with or without SR9009 (5 μM). n = 3.

(O-R) Representative images and quantification of immunofluorescence for Ki67 (**O** and **P**) and cleaved caspase 3 (CC3, **Q** and **R**) in shC and sh*TFPI2* GSC272 tumors from mice treated with or without SR9009. Scale bar, 100 μm. n = 3.

Data from multiple replicates are presented as mean ± SD. n.s., not significant, *, $P < 0.05$, ***, $P < 0.001$, one-way ANOVA test (I, J, L, N, P and R), two-way ANOVA test (G).

Supplemental Figure 4

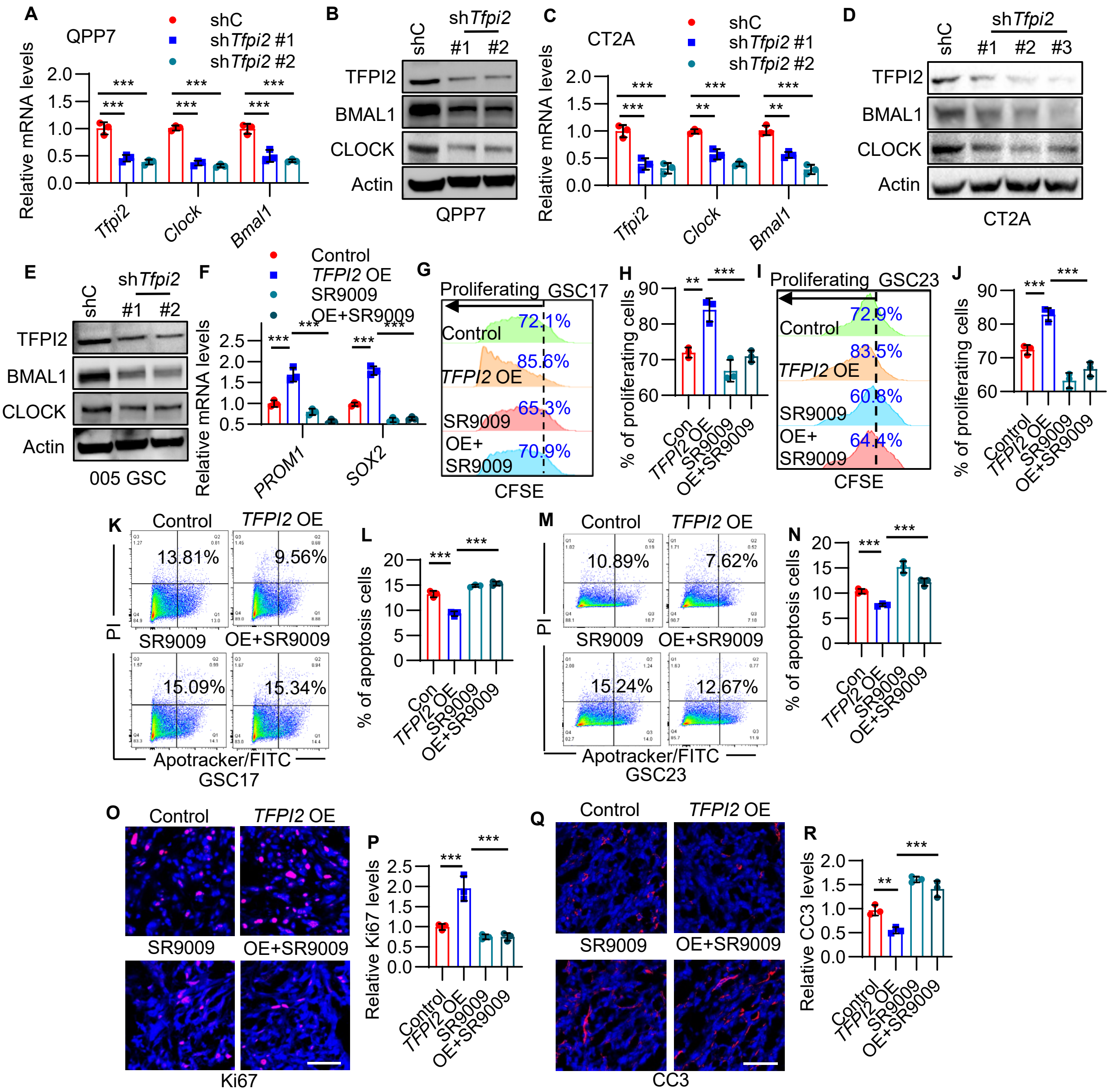

Supplemental Figure 4. The CLOCK/BMAL1 complex is regulated by TFPI2 and required for TFPI2-induced GSC proliferation and apoptosis.

(A) RT-qPCR for *Tfpi2, Clock*, and *Bmal1* in QPP7 GSCs harboring shRNA control (shC) or *TFPI2* shRNA (sh*Tfpi2*). n = 3.
(B) Immunoblots for CLOCK, BMAL1, and TFPI2 in QPP7 GSCs harboring shC or sh*Tfpi2*.
(C) RT-qPCR for *Tfpi2, Clock* and *Bmal1* in CT2A cells harboring shC or sh*Tfpi2*. n = 3.
(D and **E)** Immunoblots for CLOCK, BMAL1, and TFPI2 in CT2A cells **(D)** and 005 GSCs **(E)** harboring shC or sh*Tfpi2*.
(F) RT-qPCR for *PROM1* and *SOX2* in GSC23 harboring control or *TFPI2* overexpression (OE) treated with or without SR9009 (5 μM).
(G-J) Representative and quantification of proliferation in GSC17 **(G** and **H)** and GSC23 **(I** and **J)** harboring control or *TFPI2* OE treated with or without SR9009 (5 μM). n = 3.
(K-N) Representative and quantification of apoptosis in GSC17 **(K** and **L)** and GSC23 **(M** and **N)** harboring control or *TFPI2* OE treated with or without SR9009 (5 μM). n = 3.
(O-R) Representative images and quantification of immunofluorescence for Ki67 (**O** and **P**) and cleaved caspase 3 (CC3, **Q** and **R**) in control and *TFPI2* OE GSC272 tumors from mice treated with or without SR9009. Scale bar, 100 μm. n = 3.

Data from multiple replicates are presented as mean ± SD. **, $P < 0.01$, ***, $P < 0.001$, one-way ANOVA test (A, C, F, H, J, L, N, P and R).

Supplemental Figure 5

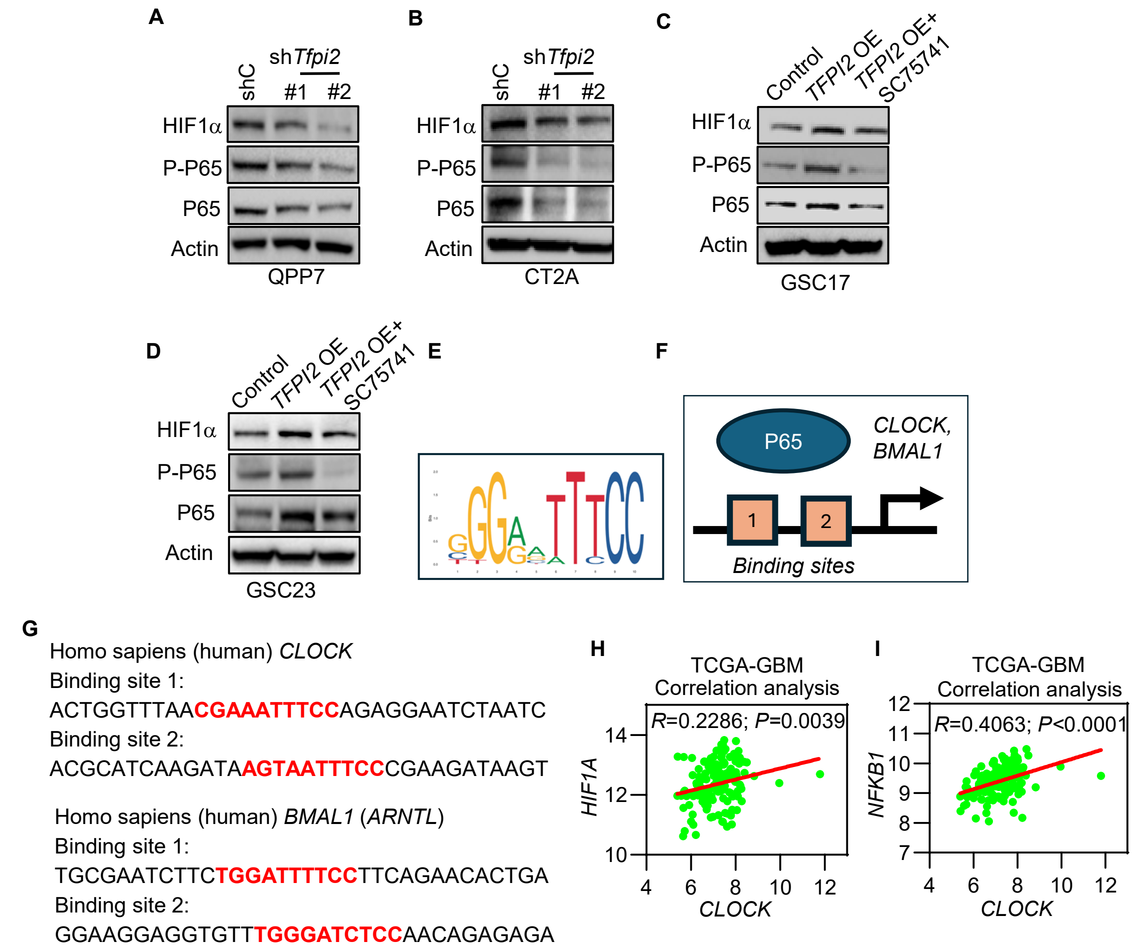

Supplemental Figure 5. TFPI2 regulates CLOCK/BMAL1 complex through HIF1α-NF-κB signaling in GSCs.

(A and **B)** Immunoblots for HIF1α, P-P65 and P65 in QPP7 GSCs **(A)** and CT2A cells **(B)** harboring shRNA control (shC) or *Tfpi2* shRNA (sh*Tfpi2*).

(C and **D)** Immunoblots for HIF1α, P-P65 and P65 in GSC17 **(C)** and GSC23 **(D)** harboring control or *TFPI2* overexpression (OE) and treated with or without P65 inhibitor SC75741 (5 μM).

(E) Binding motif of transcription factor P65.

(F) Schematic of designing ChIP-qPCR primers based on 2 potential binding sites.

(G) Schematic of the putative P65 binding motifs in the *CLOCK* and *BMAL1* promoter region in Homo sapiens.

(H and **I)** Correlation of *HIF1-A* (H) and *NFKB1* (I) with *CLOCK* in TCGA GBM patients. *R* and *P* values are shown. Pearson's correlation test.

Supplemental Figure 6

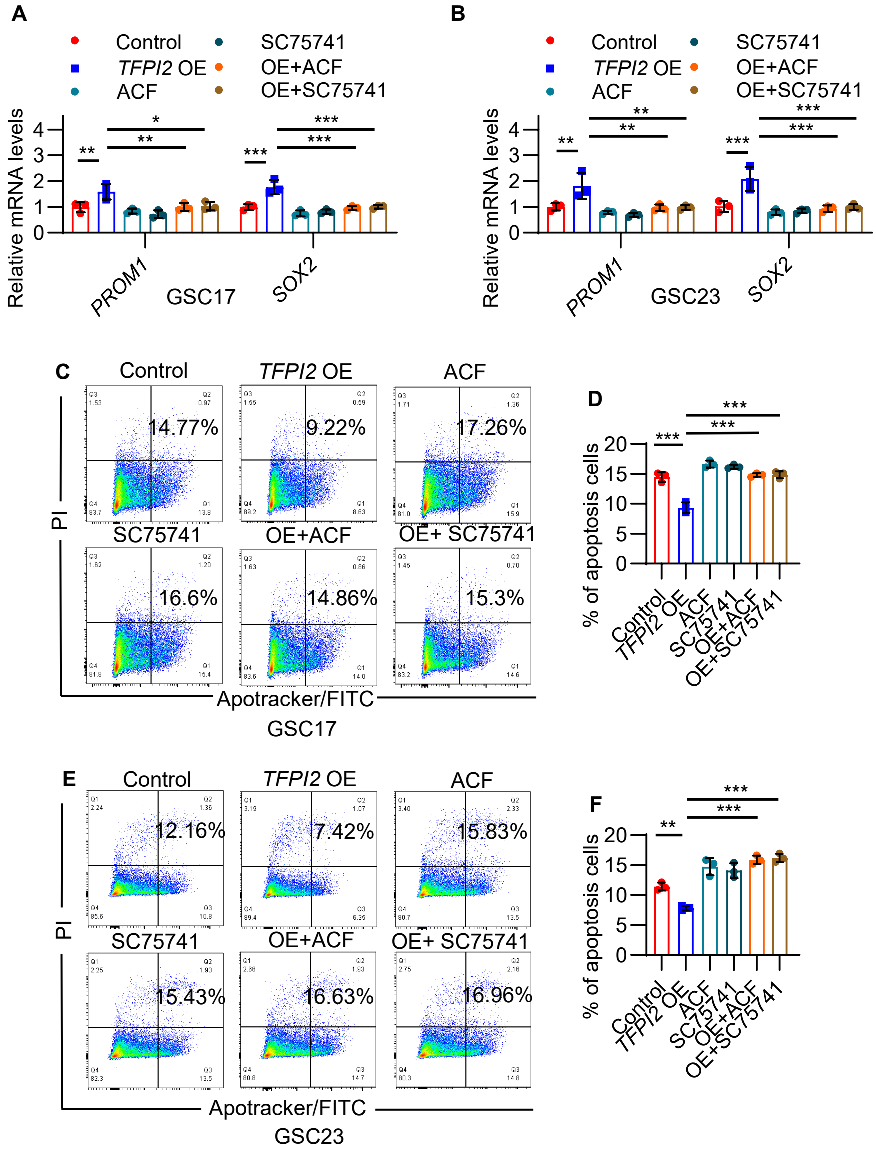

Supplemental Figure 6. HIF1α-NF-κB signaling axis mediates TFPI2-induced GSC self-renewal and apoptosis.

(A and **B)** RT-qPCR for *PROM1* and *SOX2* in GSC17 **(A)** and GSC23 **(B)** harboring control or *TFPI2* overexpression (OE) treated with or without HIF1α inhibitor ACF (2 μM) or P65 inhibitor SC75741 (5 μM). n = 3.

(C-F) Representative and quantification of apoptosis in GSC17 **(C** and **D)** and GSC23 **(E** and **F)** harboring control or *TFPI2* OE and treated with or without ACF (2 μM) or SC75741 (5 μM). n = 3.

Data from multiple replicates are presented as mean $\pm$ SD. *, $P < 0.05$, **, $P < 0.01$, ***, $P < 0.001$, One-way ANOVA test (A, B, D and F).

Supplemental Figure 7

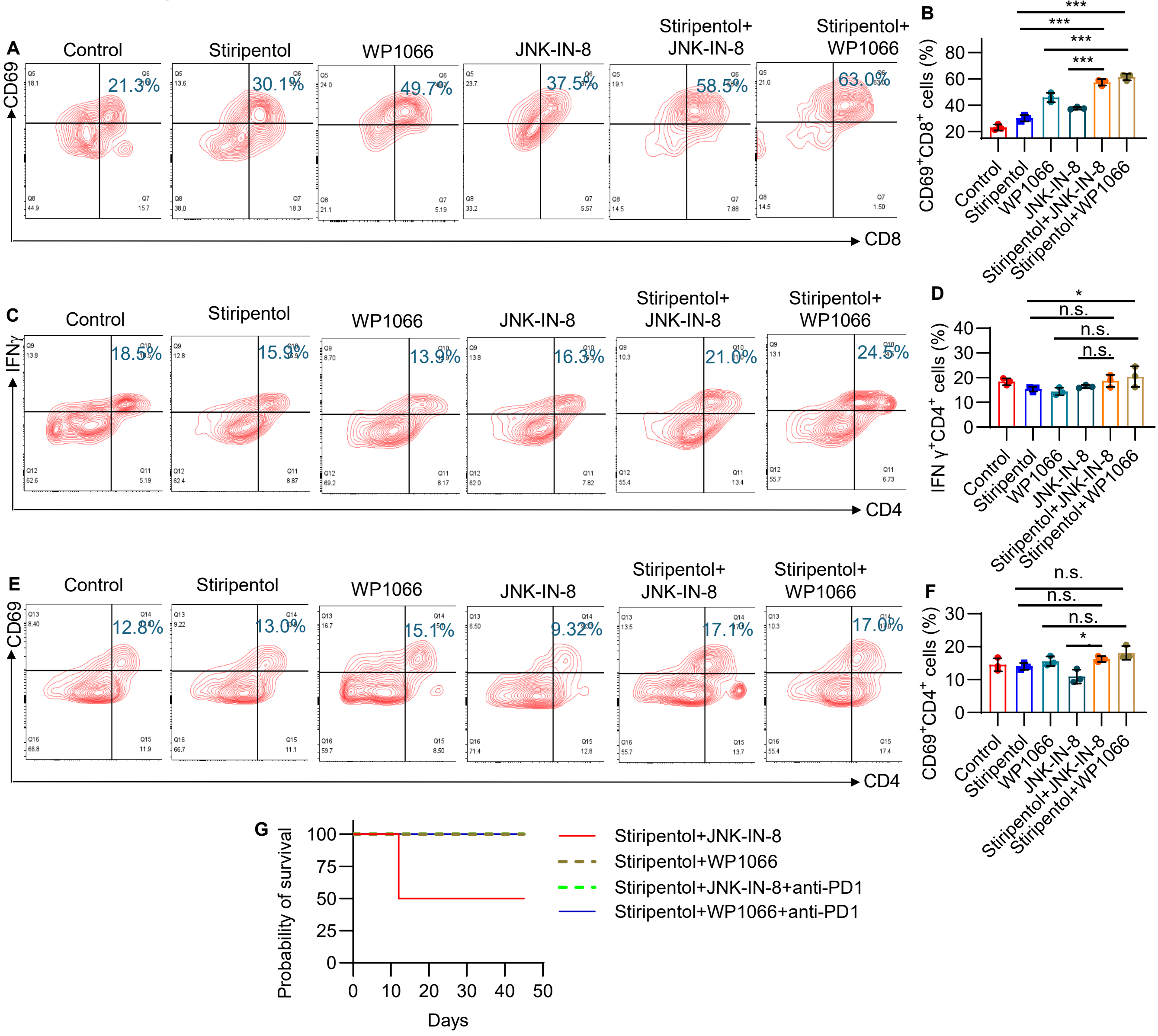

Supplemental Figure 7. Dual inhibiting the downstream pathways of TFPI2 and CLOCK activates $CD8^+$, but not $CD4^+$, T cell-mediated antitumor immunity.

(A and **B)** Representative images (**A**) and quantification (**B**) of flow cytometry for the percentage of $CD45^+CD3^+CD8^+CD69^+$ T cells (out of $CD45^+CD3^+CD8^+$ T cells) in tumors from 005 GSC-bearing mice treated with or without Stiripentol (150 mg/kg, i.p., every other day), JNK-IN-8 (30 mg/kg, i.p., daily), WP1066 (30 mg/kg, i.p., daily), and Stiripentol in combination with JNK-IN-8 or WP1066. n = 3.
(C-F) Representative images (**C** and **E**) and quantification (**D** and **F**) of flow cytometry for the percentage of $CD45^+CD3^+CD4^+IFN\gamma^+$ (**C** and **D**) or $CD45^+CD3^+CD4^+CD69^+$ (**E** and **F**) T cells (out of $CD45^+CD3^+CD4^+$ T cells) in tumors from 005 GSC-bearing mice treated with or without Stiripentol, JNK-IN-8, WP1066, and Stiripentol in combination with JNK-IN-8 or WP1066. n = 3.
(**G**) Cured mice from the double and triple therapy (from Figure 7K) were rechallenged on day 70 with 005 GSCs (2×10^5 cells/mouse).

Data from multiple replicates are presented as mean ± SD. ns, not significant, *, $P < 0.05$, ***, $P < 0.001$, One-way ANOVA test (B, D and F).

Supplementary Table 1. A list of primers used for RT-qPCR and ChIP-PCR.

Gene	Forward	Reverse
**RT-qPCR**		
Mouse *Actin*	CATGTACGTTGCTATCCAGGC	CTCCTTAATGTCACGCACGAT
Mouse *Bmal1*	CGGCCCACAAACTGGACAA	AAAGCTCTGTCATGCCTCTCA
Mouse *Clock*	ATGGTGTTTACCGTAAGCTGTAG	CTCGCGTTACCAGGAAGCAT
Mouse *Prom1*	GTTGAGACTGTGCCCATGAAA	GACGGGCTTGTCATAACAGGA
Mouse *Sox2*	GCGGAGTGGAAACTTTTGTCC	CGGGAAGCGTGTACTTATCCTT
Mouse *Tfpi2*	GTGGGCTCCGTTCTTGGTC	AAGCAGCCTCCATAGTTGAATC
Human *BMAL1*	TGGCGCGTAAACTGGACAAA	CCTCTTTCACATCCAACCACAAA
Human *CLOCK*	AGAACTTGGCATTGAAGAGTCTC	GTCAGACCCAGAATCTTGGCT
Human *GAPDH*	GGAGCGAGATCCCTCCAAAAT	GGCTGTTGTCATACTTCTCATGG
Human *PROM1*	AGTCGGAAACTGGCAGATAGC	GGTAGTGTTGTACTGGGCCAAT
Human *SOX2*	TGCAGCATGTCCTACTCGCAG	GAGGAAGAGGTCACCACAGGG
Human *TFPI2*	CTGGGGCTGTCGATTCTGC	TCTCCGCGTTATTTCCTGTTG
**ChIP-qPCR**		
**Human *BMAL1* #1-1**	AGGTAGTAGTGGAAGGAGGTGT	CCCTCATGTGCACCTGTTTT
**Human *BMAL1* #1-2**	AGGTAGTAGTGGAAGGAGGTG	ATGTGCACCTGTTTTTCCCTG
**Human *BMAL1* #2-1**	TTAGAGTCAGCCCACTGCTCC	TCAAAGCCTCTATACCACACCA
**Human *BMAL1* #2-2**	GTTAGAGTCAGCCCACTGCTCC	TCAAAGCCTCTATACCACACCAG
**Human *CLOCK* #1-1**	TGTCAGTCGGGACTCTTGAT	CTGAGAAAGAAGGCAACACGG
**Human *CLOCK* #1-2**	TGTCAGTCGGGACTCTTGA	GACAGGTTTGGCCTAAAGACA
**Human *CLOCK* #2-1**	ATGTGGACCAAATGTGCCTT	CGTTGGAACAACGTGTGACT
**Human *CLOCK* #2-2**	ATGTGCTTTCTCATGTGGACC	CTCGTTGGAACAACGTGTGAC
