# Supplementary material for: Symbiotic exclusivity between CLOCK and TFPI2 drives stemness and immunosuppression in glioblastoma models
Source: J Clin Invest. 2026 Mar 17;136(10):e199056. doi: 10.1172/JCI199056 (PMC13178667; doi:10.1172/JCI199056)

Full unedited blots for Figure 1

Figure.1K

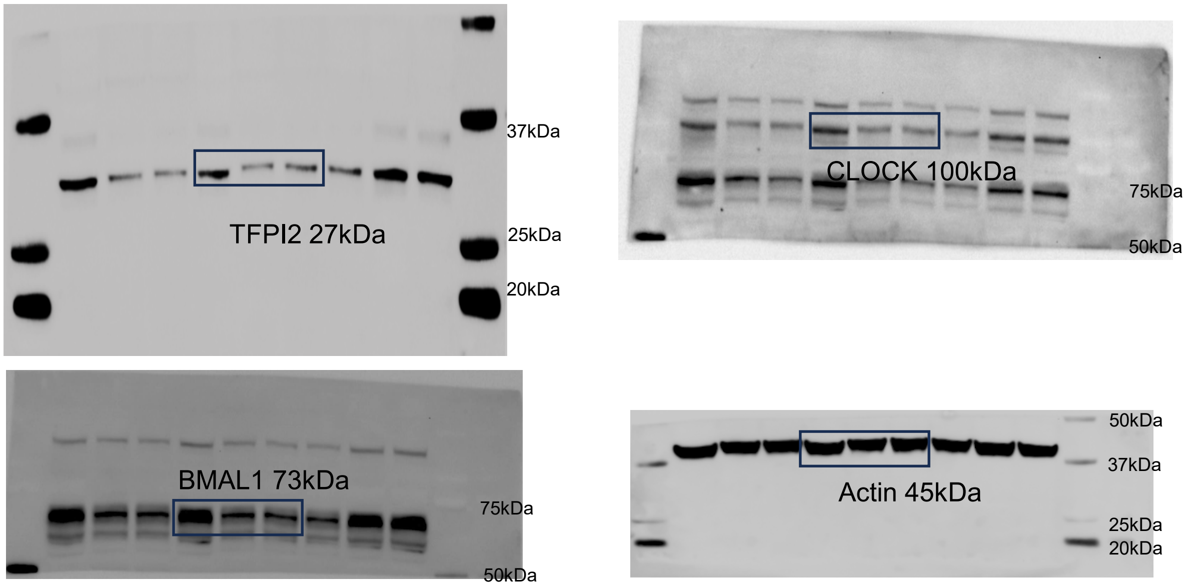

Figure.1L

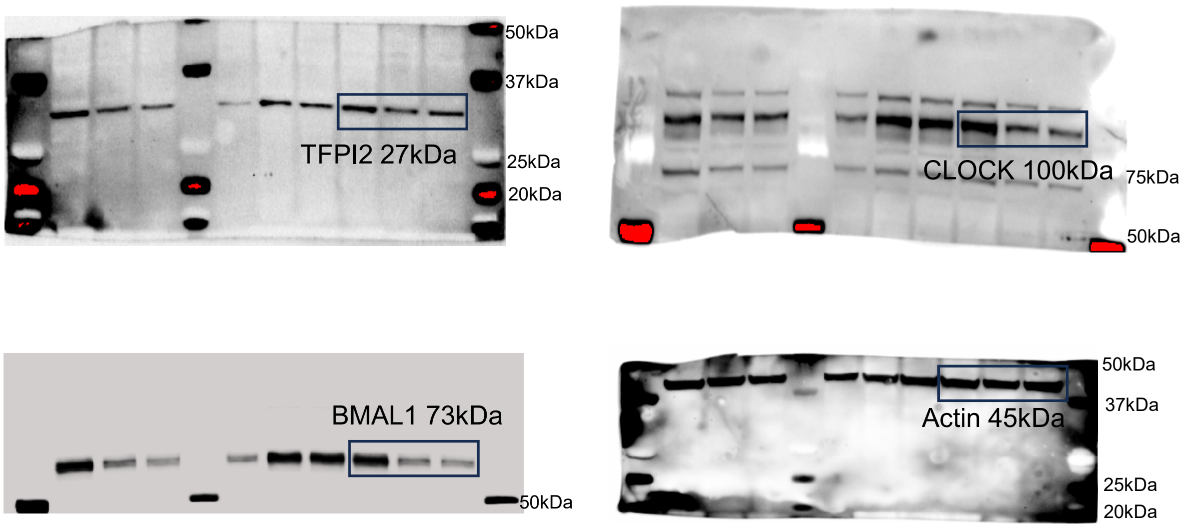

Full unedited blots for Figure 1

Figure.1M

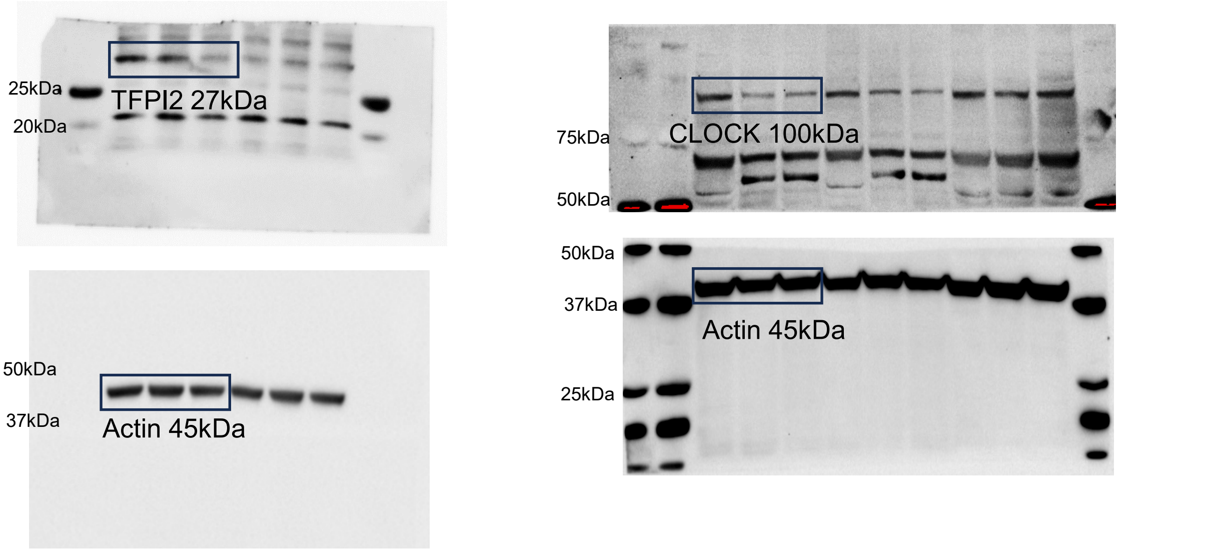

Figure.1N

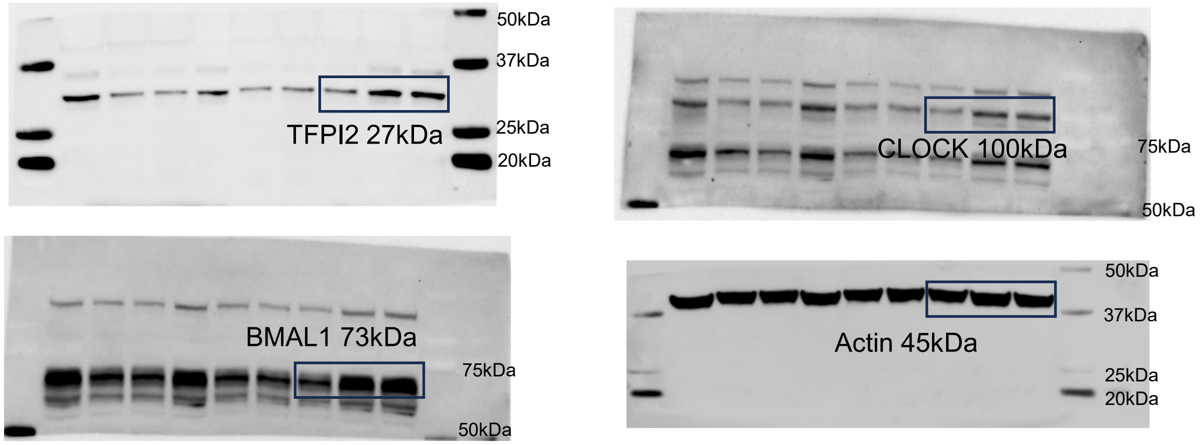

Figure.1O

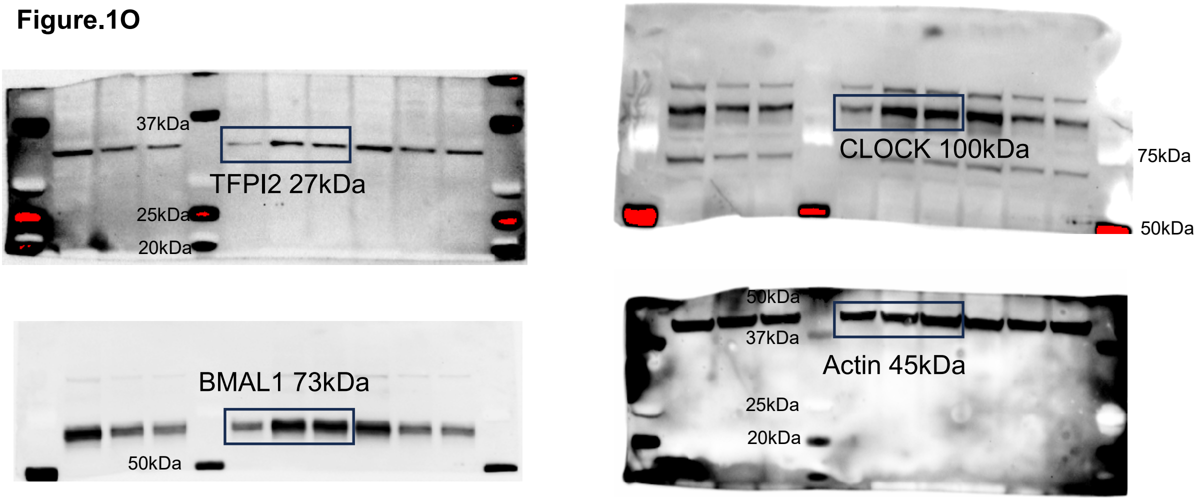

Full unedited blots for Figure 2

Figure.2A

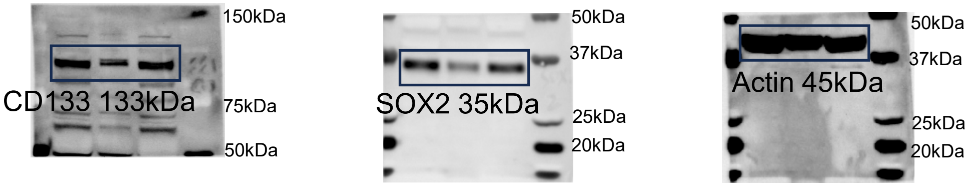

Figure.2B

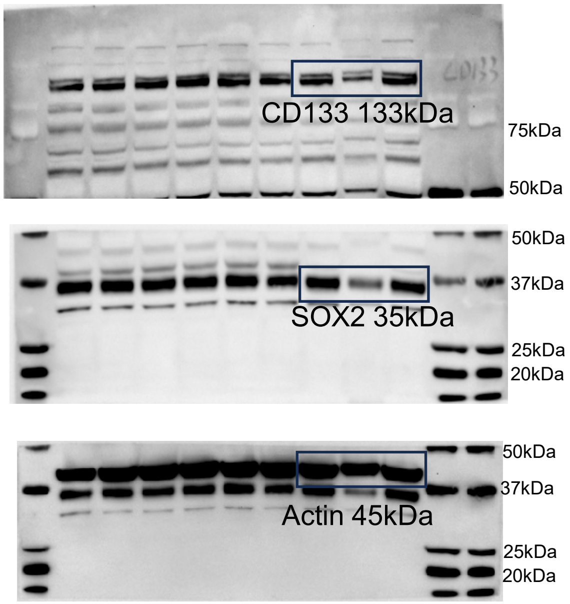

Figure.2N

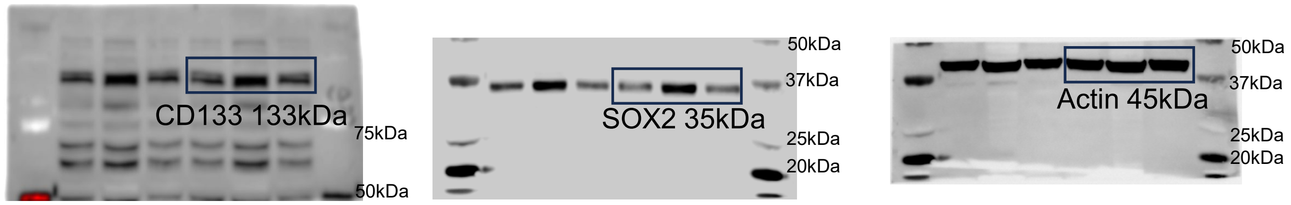

Figure.2O

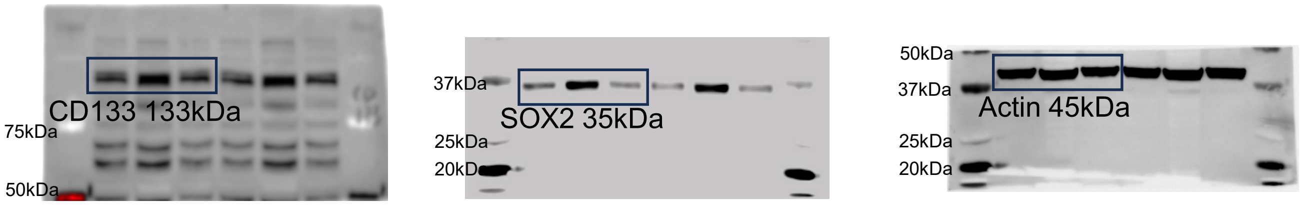

Full unedited blots for Figure 4

Figure.4C

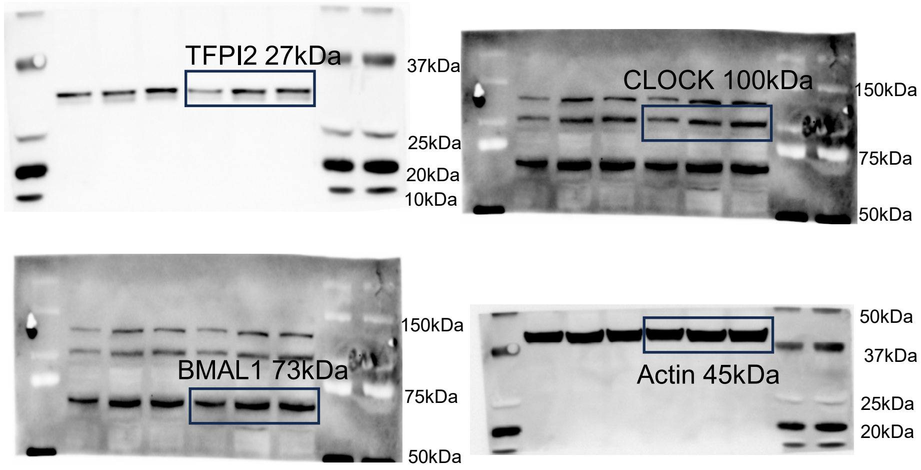

Figure.4D

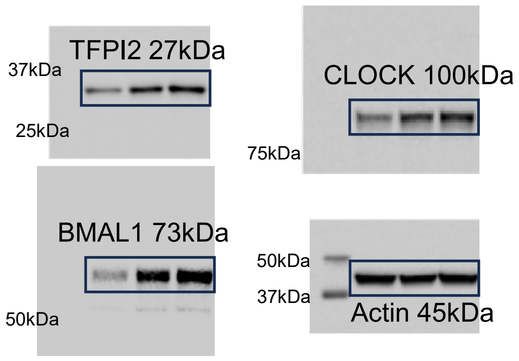

Figure.4F

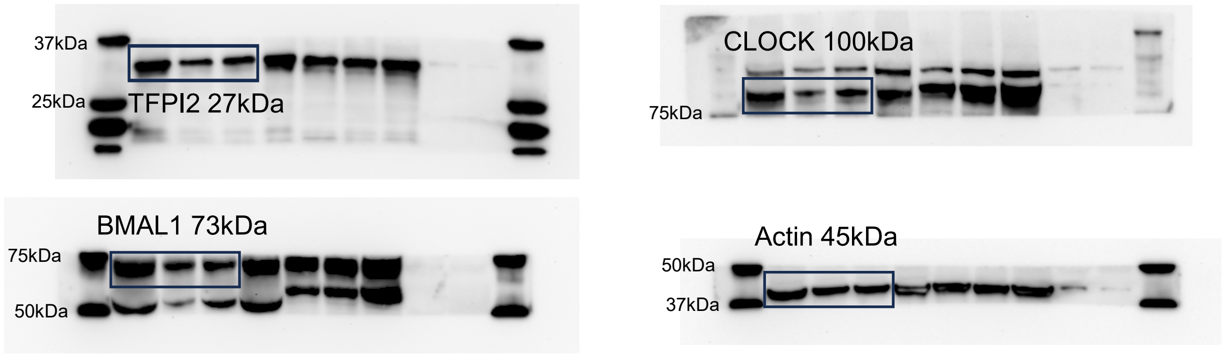

Figure.4G

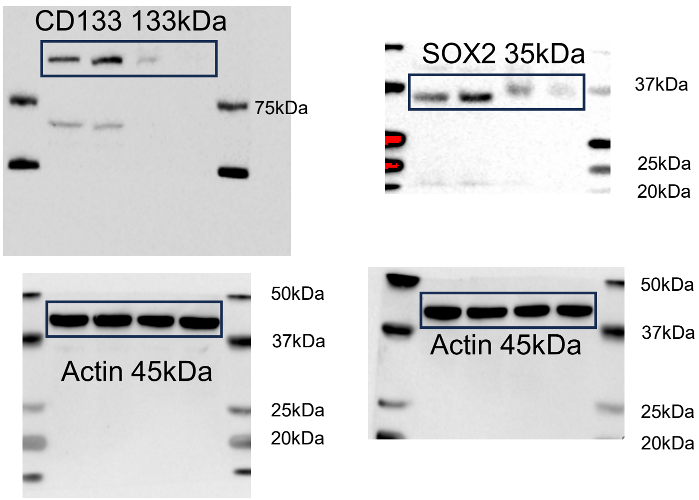

Figure.4H

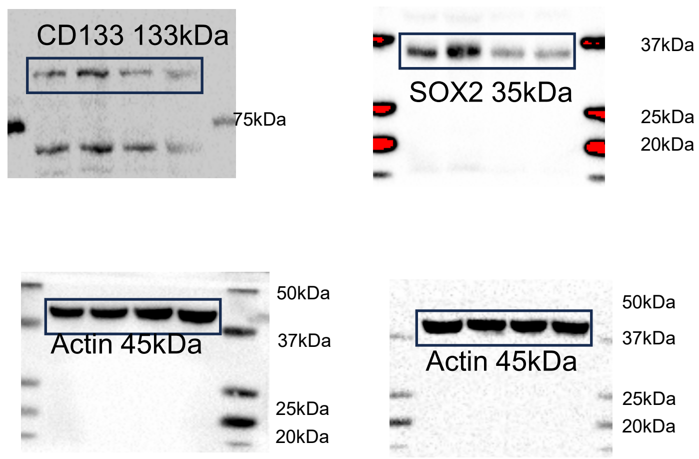

Full unedited blots for Figure 5

Figure.5B

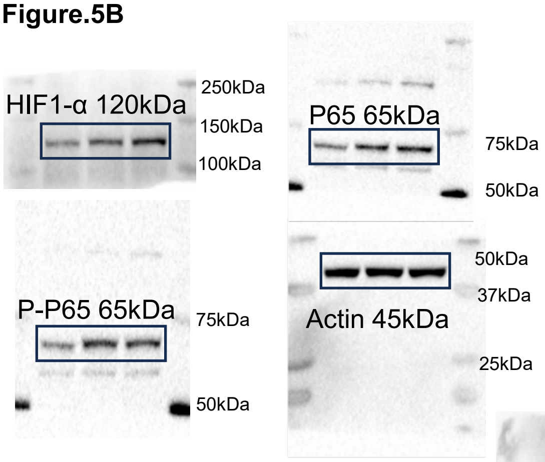

Figure.5C

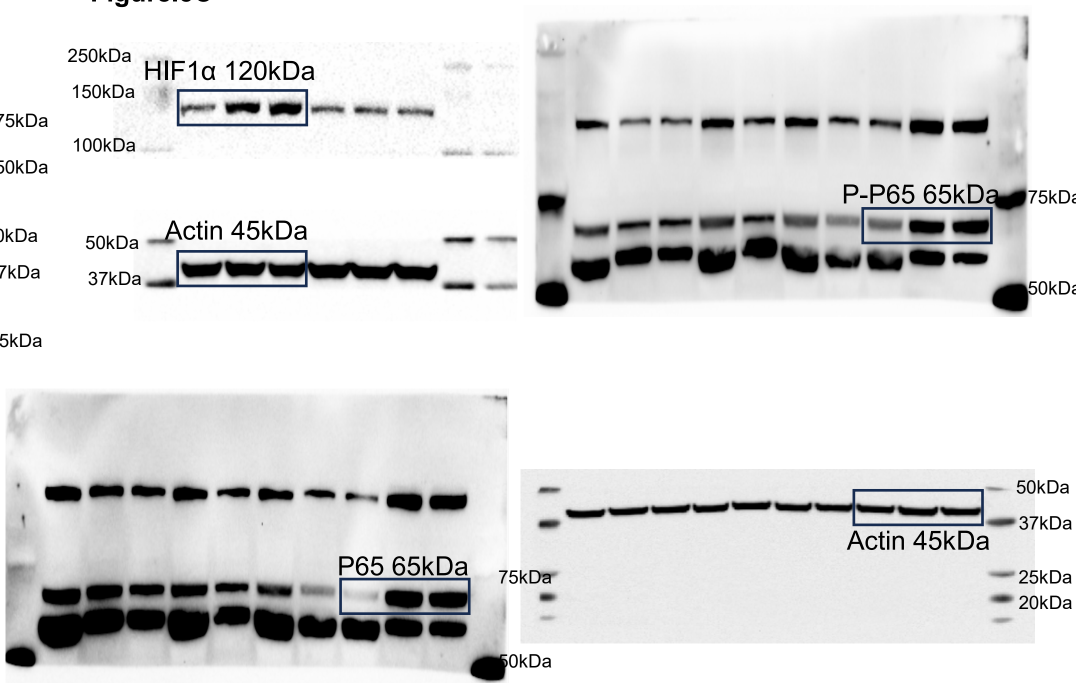

Figure.5D

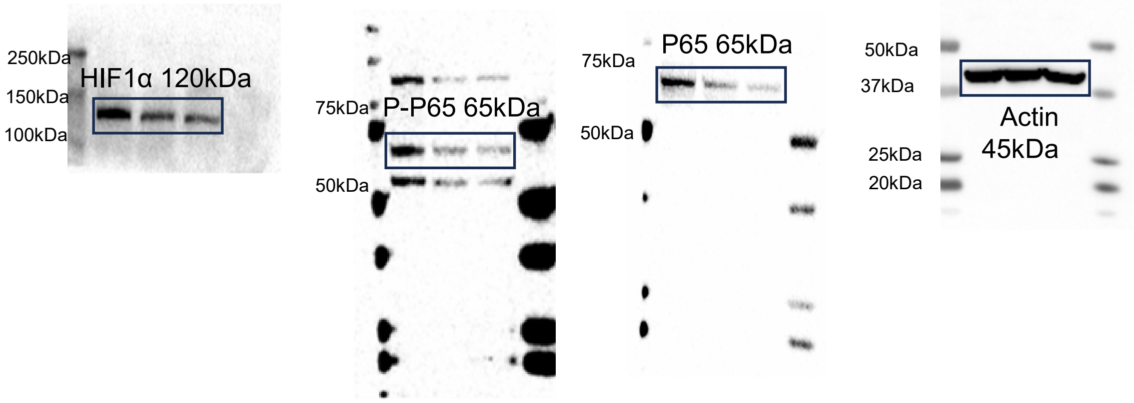

Figure.5E

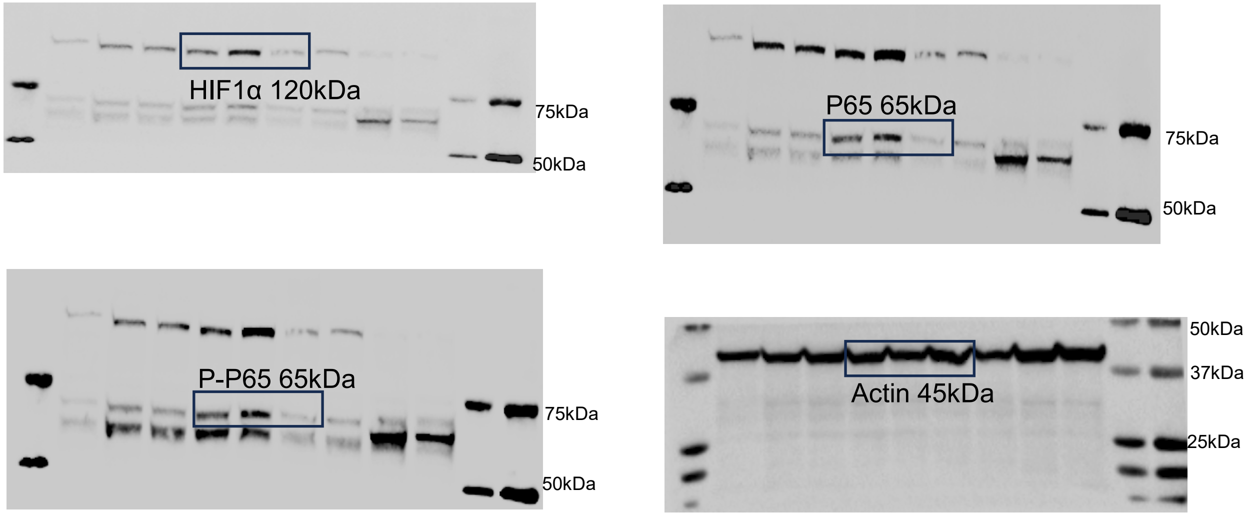

Full unedited blots for Figure 5

Figure.5F

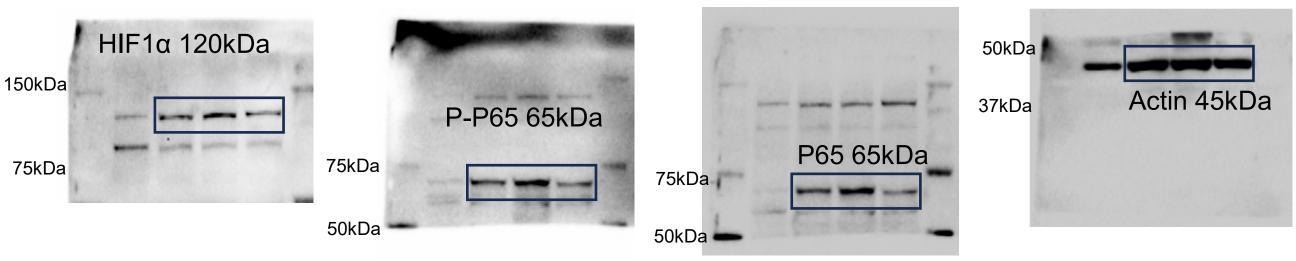

Figure.5M

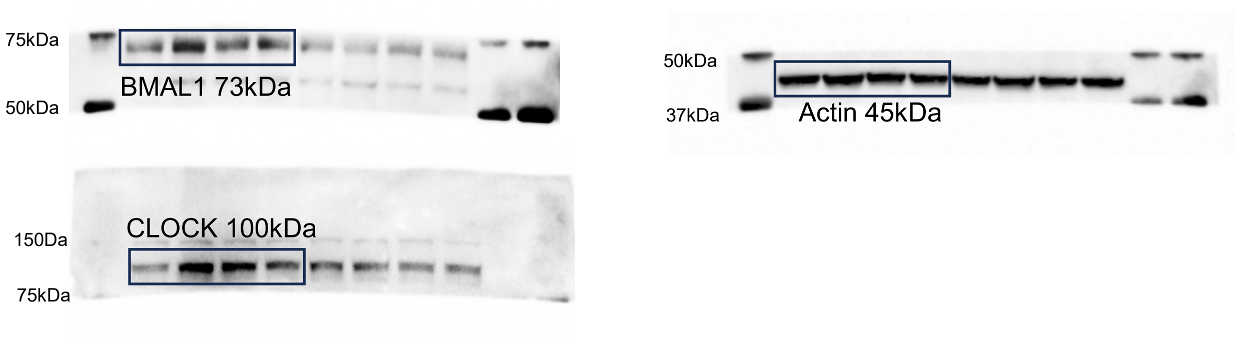

Figure.5N

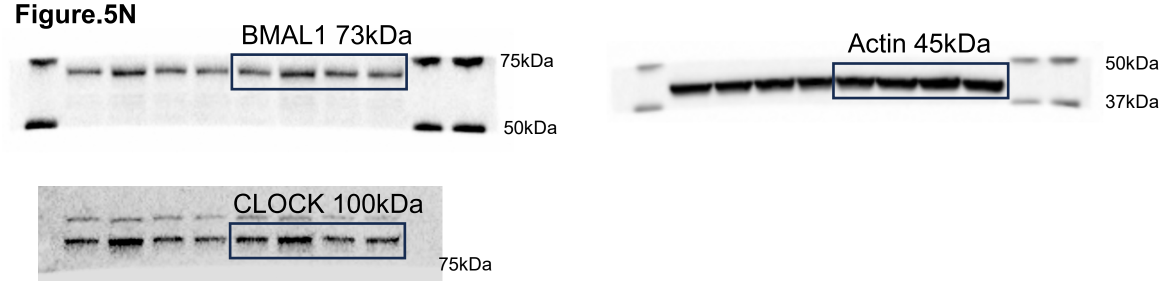

Figure.5O

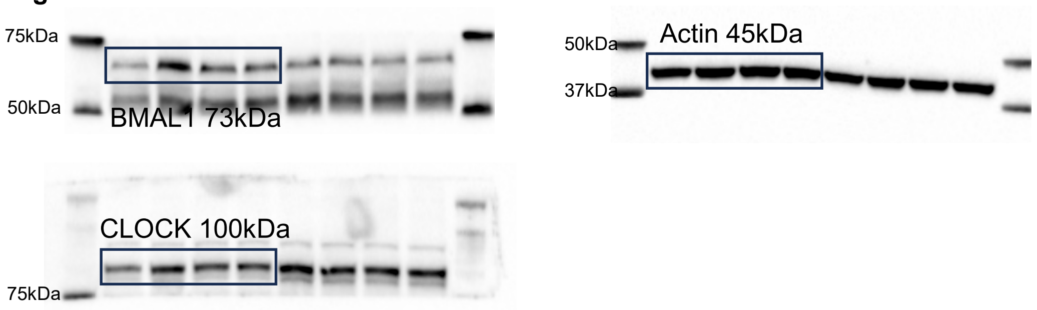

Figure.5P

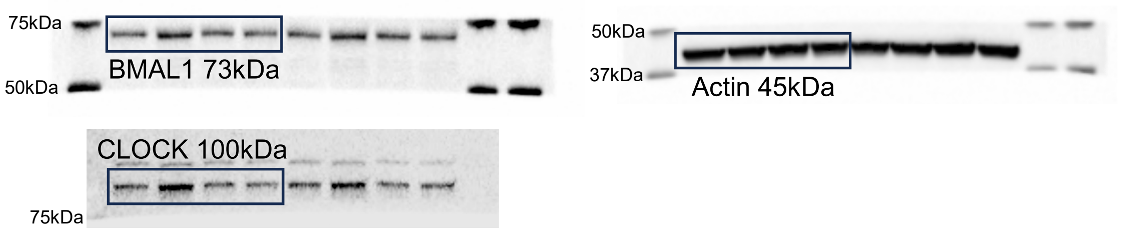

Full unedited blots for Figure 6

Figure.6A

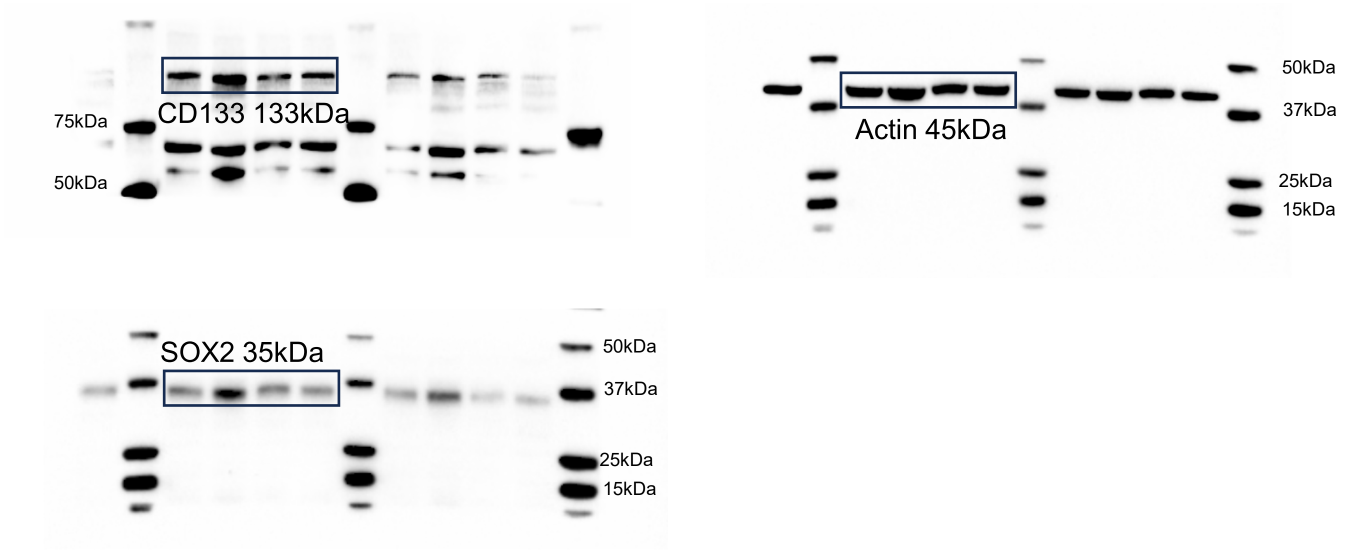

Figure.6B

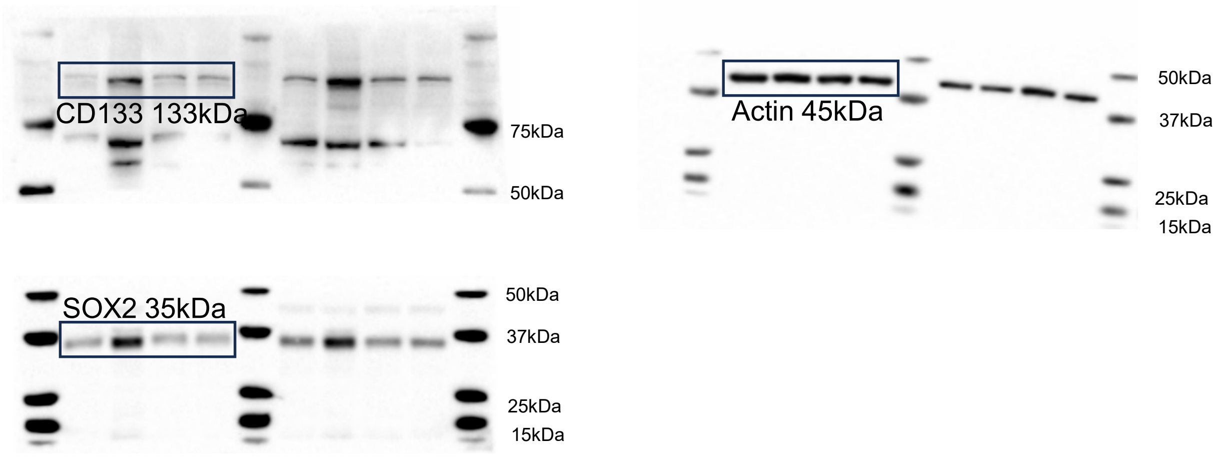

Full unedited blots for Figure 6

Figure.6C

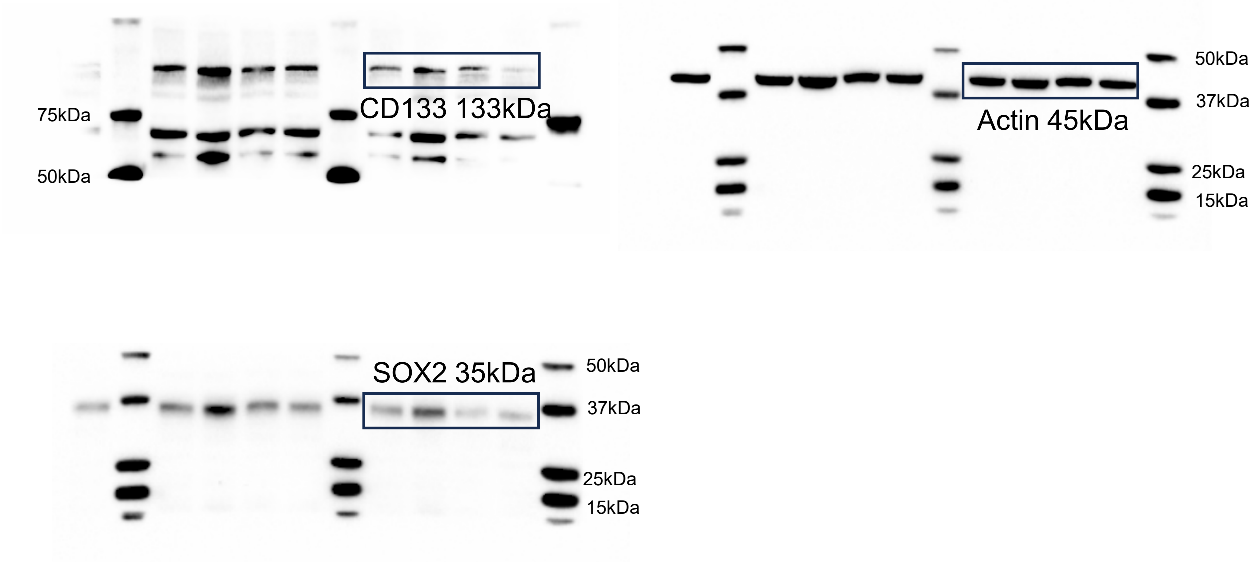

Figure.6D

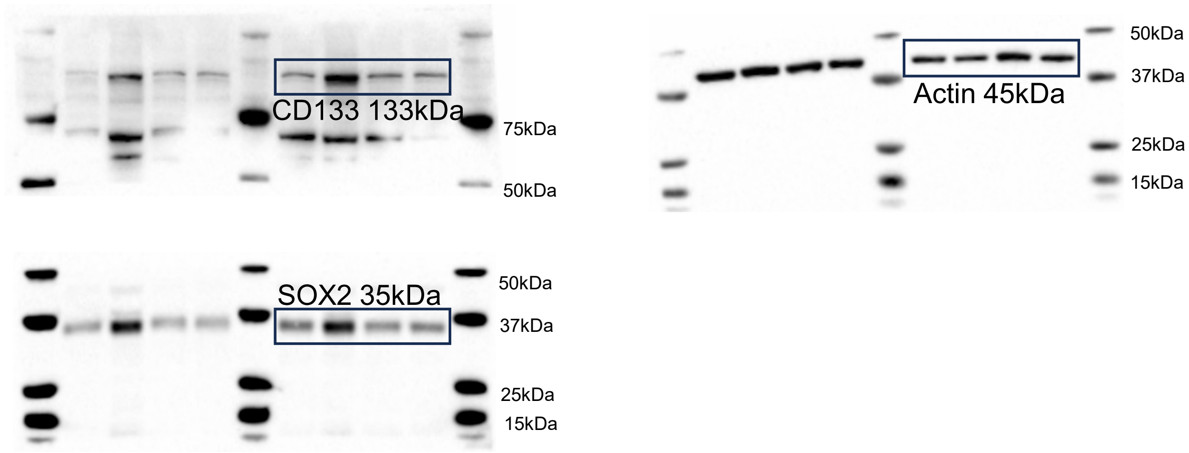

Full unedited blots for Figure S1

Figure.S1E

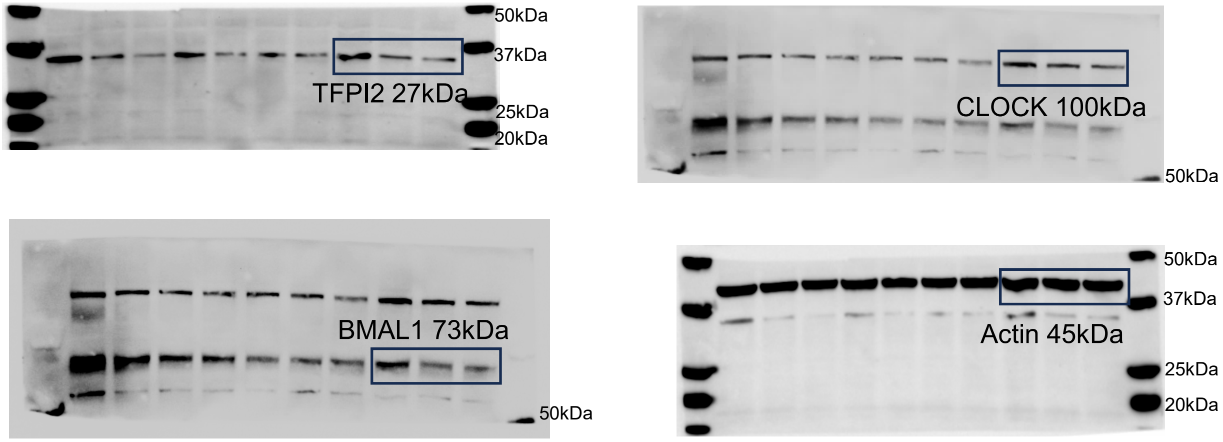

Figure.S1F

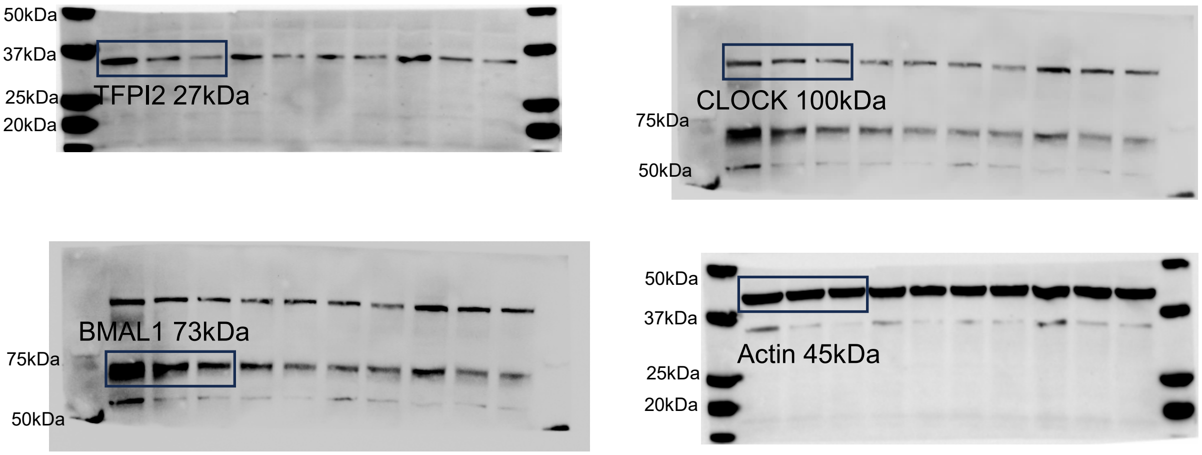

Figure.S1G

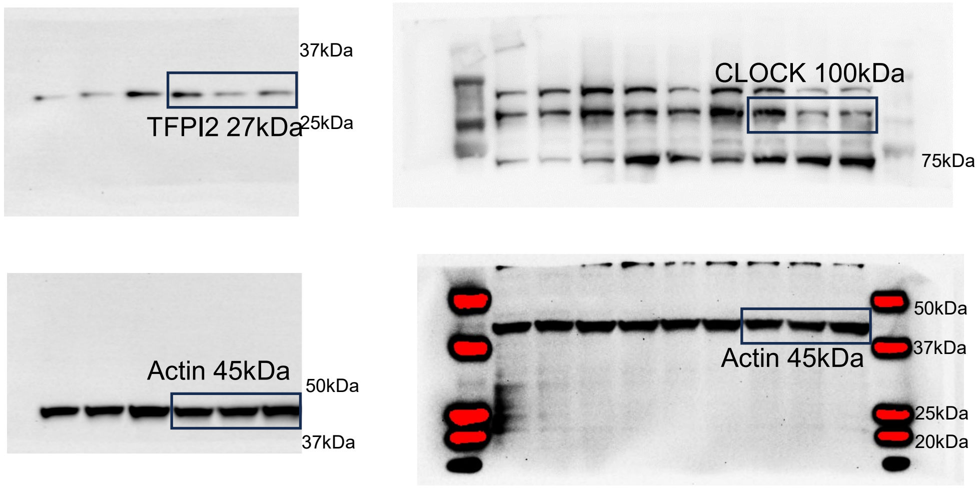

Full unedited blots for Figure S1

Figure.S1H

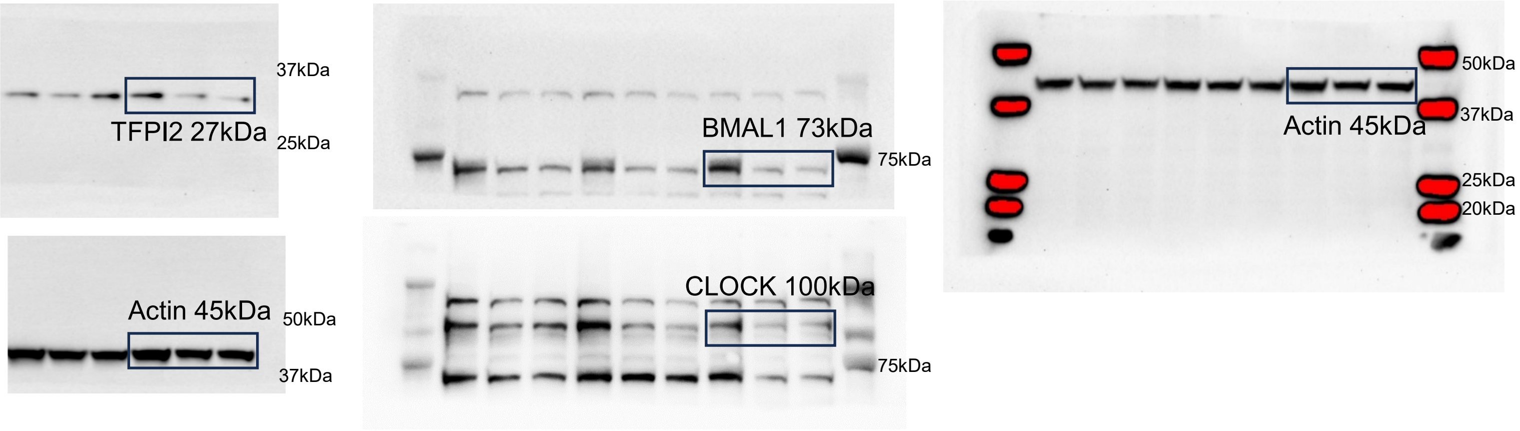

Figure.S1I

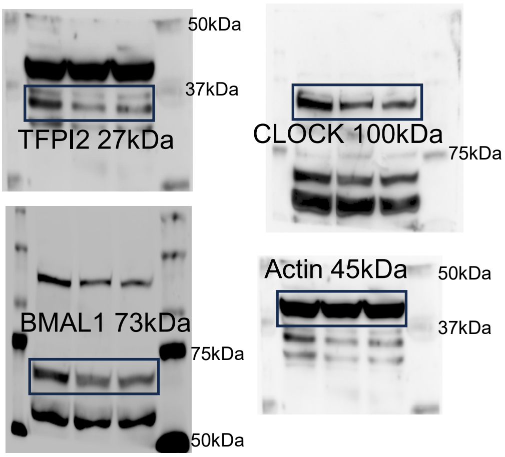

Figure.S1J

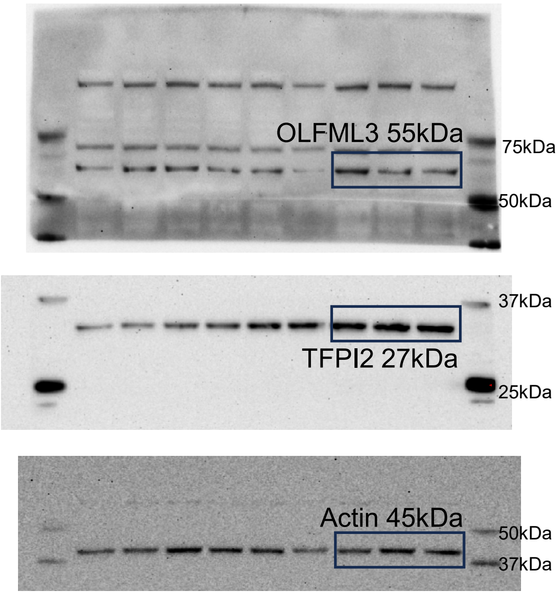

Full unedited blots for Figure S2

Figure.S2A

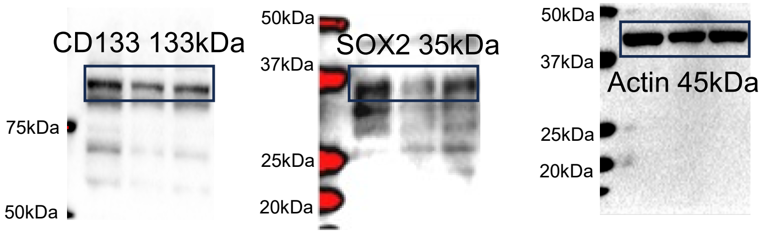

Figure.S2B

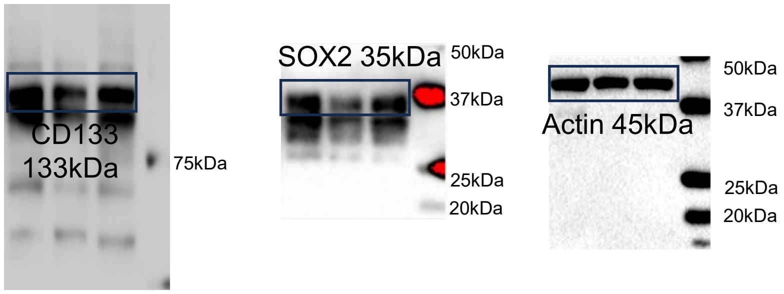

Figure.S2C

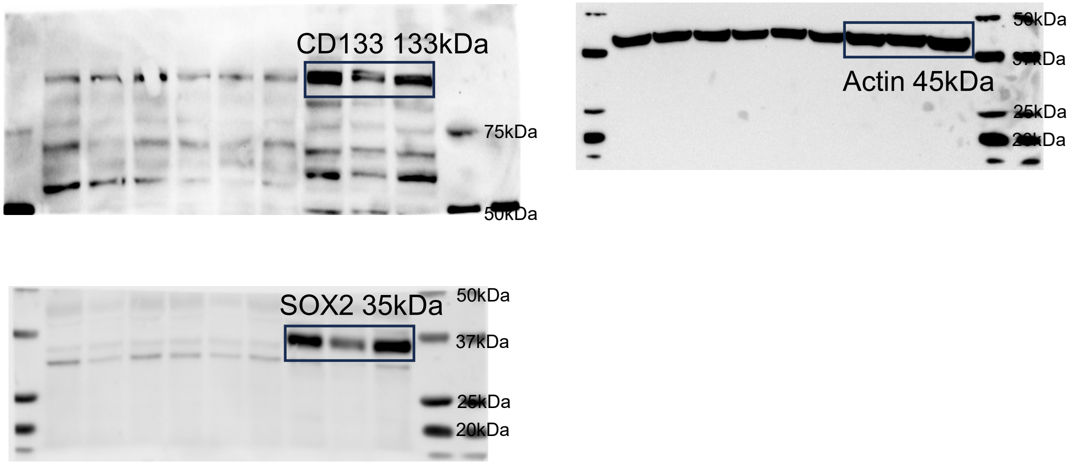

Full unedited blots for Figure S3

FigureS3E

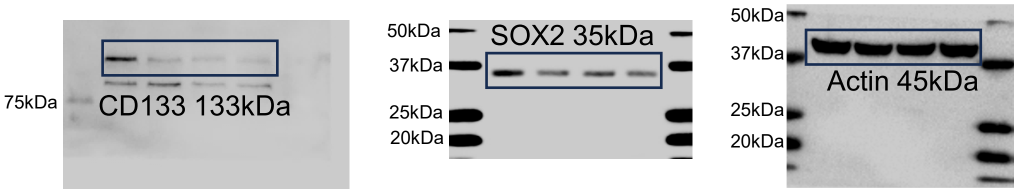

Figure.S3F

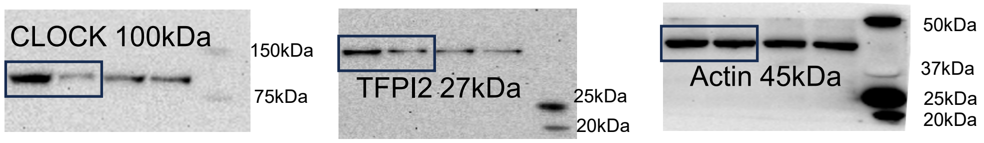

Full unedited blots for Figure S4

Figure.S4B

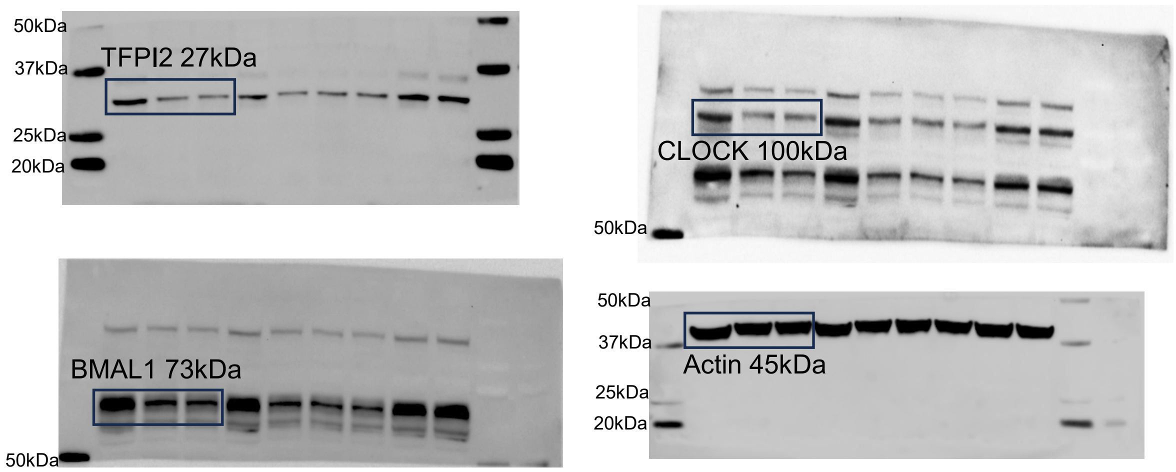

Figure.S4D

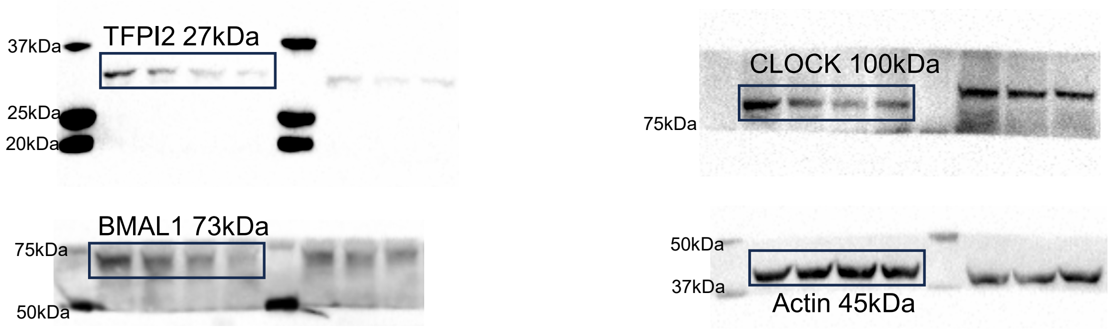

Figure.S4E

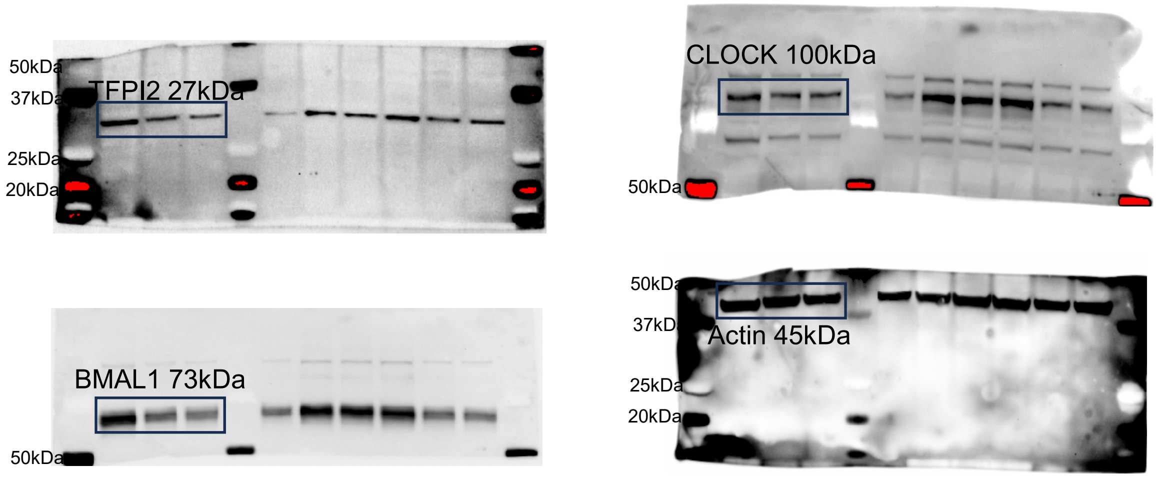

Full unedited blots for Figure S5

Figure.S5A

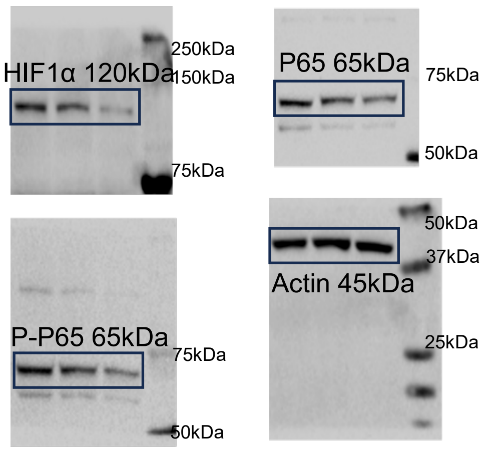

Figure.S5B

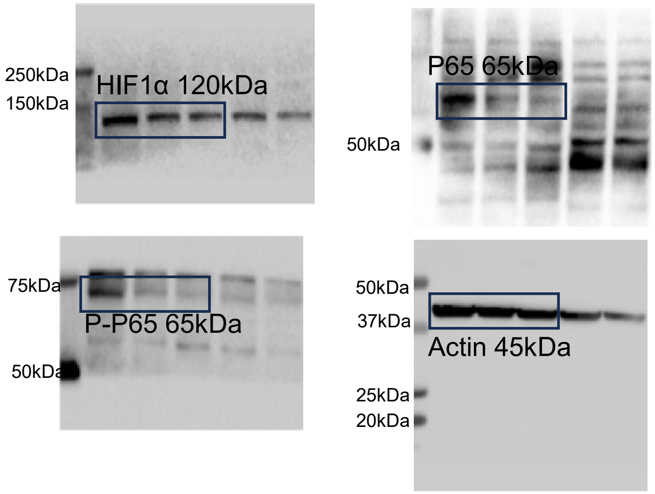

Figure.S5C

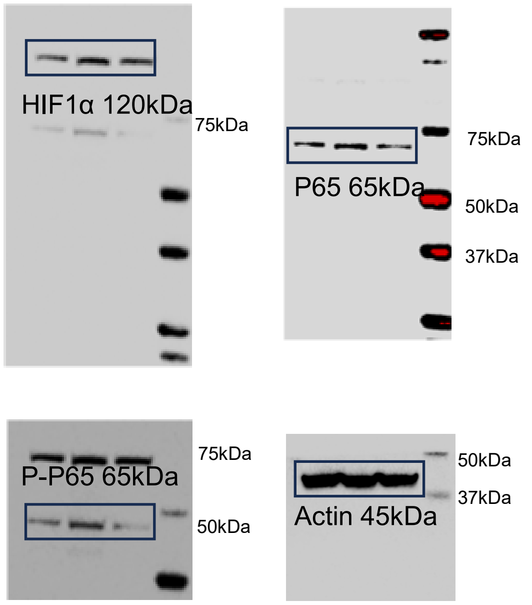

Figure.S5D

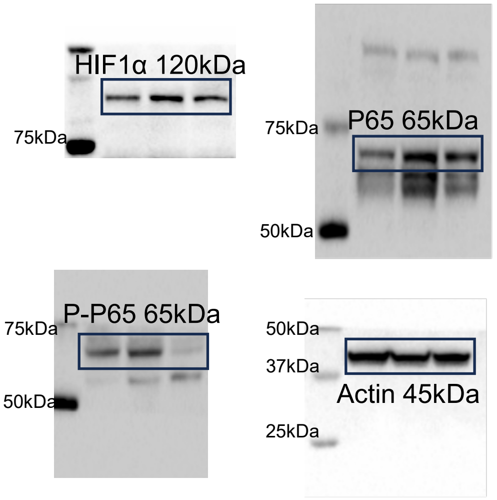

Supplement: Unedited blot and gel images [file jci-136-199056-s052.pdf]
